# Pathogen-Specific Binding Soluble Down Syndrome Cell Adhesion Molecule (Dscam) Regulates Phagocytosis *via* Membrane-Bound Dscam in Crab

**DOI:** 10.3389/fimmu.2018.00801

**Published:** 2018-04-18

**Authors:** Xue-Jie Li, Lei Yang, Dan Li, You-Ting Zhu, Qun Wang, Wei-Wei Li

**Affiliations:** Laboratory of Invertebrate Immunological Defense and Reproductive Biology, School of Life Sciences, East China Normal University, Shanghai, China

**Keywords:** membrane-bound Down syndrome cell adhesion molecule, soluble Down syndrome cell adhesion molecule, specific-binding, pathogen, phagocytosis, *Eriocheir sinensis*

## Abstract

The Down syndrome cell adhesion molecule (Dscam) gene is an extraordinary example of diversity that can produce thousands of isoforms and has so far been found only in insects and crustaceans. Cumulative evidence indicates that Dscam may contribute to the mechanistic foundations of specific immune responses in insects. However, the mechanism and functions of Dscam in relation to pathogens and immunity remain largely unknown. In this study, we identified the genome organization and alternative Dscam exons from Chinese mitten crab, *Eriocheir sinensis*. These variants, designated *Es*Dscam, potentially produce 30,600 isoforms due to three alternatively spliced immunoglobulin (Ig) domains and a transmembrane domain. *Es*Dscam was significantly upregulated after bacterial challenge at both mRNA and protein levels. Moreover, bacterial specific *Es*Dscam isoforms were found to bind specifically with the original bacteria to facilitate efficient clearance. Furthermore, bacteria-specific binding of soluble *Es*Dscam *via* the complete Ig1–Ig4 domain significantly enhanced elimination of the original bacteria *via* phagocytosis by hemocytes; this function was abolished by partial Ig1–Ig4 domain truncation. Further studies showed that knockdown of membrane-bound *Es*Dscam inhibited the ability of *Es*Dscam with the same extracellular region to promote bacterial phagocytosis. Immunocytochemistry indicated colocalization of the soluble and membrane-bound forms of *Es*Dscam at the hemocyte surface. Far-Western and coimmunoprecipitation assays demonstrated homotypic interactions between *Es*Dscam isoforms. This study provides insights into a mechanism by which soluble Dscam regulates hemocyte phagocytosis *via* bacteria-specific binding and specific interactions with membrane-bound Dscam as a phagocytic receptor.

## Introduction

Although the specializations of the immune system that define adaptive immunity were believed to be unique to gnathostomes (jawed vertebrates) (1, 2), a possible alternative adaptive immune system has recently been identified in lamprey (3) and arthropods (4, 5). It has been reported that highly specific immune protection to certain pathogens can be transferred from mother to offspring in crustacean and insects (6–10), and specific life-long immunological memory can be sustained, providing protection against the pathogen responsible for the original infection (11). Thus, accumulating evidence suggests that the arthropod immune system has greater complexity than previously appreciated.

The vertebrate immune system discriminates between a plethora of microbes through a diverse repertoire of pattern-recognition receptors generated by V(D)J recombination and somatic hyper-mutation of the antibody immunoglobulin (Ig) domains. By contrast, although arthropods do not produce antibodies, approximately 150 Ig domain protein-encoding genes have been identified in some insect genomes (12–14). Most of these genes have been studied with regard to neuronal guidance, although the implications for immunity have been investigated more recently (12–14). The Down syndrome cell adhesion molecule gene, *Dscam*, which is a member of the arthropod Ig superfamily, can potentially produce 38,016 different forms through alternative splicing of exons in *Drosophila melanogaster* (14). Moreover, membrane-bound and soluble forms of Dscam (mDscam and sDscam), which are distinguished only by the presence or absence of transmembrane and cytoplasmic tail regions, have also been identified in hemocyte and cell-free hemolymph (13), respectively. Therefore, it is conceivable that a large protein isoform repertoire with the potential for recognizing diverse ligands and epitopes could be generated in crustaceans and insects.

Limited research on the Dscam-mediated immune responses has been published (13, 15–20), although these studies have provided pioneering insights into the specific arthropod immune responses. Studies in mosquito demonstrated that alternative splicing of Dscam can generate pathogen-specific isoforms in response to immune challenge (16). Moreover, Dscam knockdown was shown to impair the capacity of hemocytes to phagocytose bacteria in *D. melanogaster*, while Dscam in hemocytes of *D. melanogaster* (13) and *Anopholes gambiae* (16) bind *Escherichia coli* and potentially acts as both a phagocytic receptor and subsequently as an opsonin. The crystal structures of the two Dscam isoforms revealed two distinct surface epitopes (epitope I and epitope II) that are composed of hypervariable amino-acid residues and conserved residues, allowing a given Dscam isoform to form a homodimer and retain the ability to potentially recognize, opsonize, and crosslink pathogens (21, 22). Epitope I sequences is the N-terminal segments of both exon 4 and exon 6, which engaged in homophilic binding specificity, whereas epitope II is the C-terminal segments of both exon 4 and exon 6, which was hypothesized to bind to non-Dscam ligands, e.g., pathogens, due to the apparently faster-evolving sequence variability that would be consistent with immune receptor adaptations to dynamic alterations in host–pathogen interactions (21). These findings provide exciting evidence that Dscam may mediate distinct functions in the innate immune system of arthropods *via* the preferential use of splice variants post-infection and also highlight the potential importance for epitope I and II in Dscam-mediated immune reactions.

Chinese mitten crab, *Eriocheir sinensis*, is one of the most important economic aquaculture crustacean species in southeast Asia. In recent years, the frequent outbreaks of diseases have given rise to decreased production and enormous economic loss. Most of these diseases in *E. sinensis* are usually caused by different bacteria, for this reason, the study of the pattern-recognition receptors would help to reveal the pathogenesis and expand the knowledge on arthropods’ Dscam functions. In this study, we raised the hypothesis that pathogen-induced particular soluble Dscam isoforms specific binding with the original bacteria *via* epitope II, and then interact with membrane-bound Dscam that share the same extracellular regions *via* epitope I to promote phagocytosis. To test that hypothesis, the genome structure of *Eriocheir sinensis Dscam* (*EsDscam*) was revealed and every alternative spliced exon was confirmed. After that, the mRNA and protein expression level post bacteria stimulation were analyzed, the candidate alternatively spliced exons that may contribute to bacteria-specific binding were captured by testing the bacteria-binding activity of epitope II, and confirmed by test recombinant protein mediated bacteria-specific binding activity *in vitro* and bacteria clearance *in vivo*. Furthermore, soluble *EsDscam* regulated hemocyte phagocytosis and the role of specific membrane-bound *EsDscam* in this process were tested. This study provides novel data testing the role of epitope II in bacteria-specific binding, and epitope I-mediated interaction between membrane-bound and soluble Dscam. To the best of our knowledge, these are important and as-of-yet unanswered questions in Dscam research, and this study will benefit for better understanding the previous unassured alternatively spliced genes mediated sophisticated immune functions in arthropods.

## Materials and Methods

### Animals

All animal experiments were performed according to the protocol approved by the East China Normal University Animal Care and Use Committee (Protocol license number: AR2012/12017) and in direct accordance with Ministry of Science and Technology of the People’s Republic of China on animal care guidelines. Healthy crabs (100 ± 20 g each, adult, non-antibiotic, or antifungal feed ground) were obtained from a local aquatic farm in Shanghai, China. After transfer to the laboratory, the crabs were maintained in filtered and aerated freshwater with abundance of oxygen, and fed with a commercial formulated diet (KangDa NO.183 diets for the adult crabs, Huai’an KangDa Feed Co., Ltd., Huai’an City, Jiangsu Province, China) daily which contains no antibiotics. Crabs were acclimated for a week at 22°C before use and 5% was randomly detected by PCR using TaKaRa 16S rDNA Bacterial Identification PCR Kit to ensure that the crab were free of *Staphylococcus aureus* and *Vibrio parahemolyticus*.

### Genomic Sequencing, Assembly, and Annotation

Multiple paired-end and mate-pair libraries were constructed, with a spanning size range of 180 bp to 20 kb. All libraries were sequenced on the Illumina HiSeq 2000 and PacBio platforms. After filtering out the adaptor sequences, low quality reads and duplicate reads, a total of 439.3 Gb of data were retained for assembly. The draft assembly was evaluated using transcriptome data and comparing the distributions of GC content across the whole genomes of primates, as well as by mapping the high-quality reads from paired-end libraries with short insert sizes to the scaffolds using the Burrows-Wheeler Aligner (unpublished). Subsequently, the *EsDscam* gene sequence was captured from *E. sinensis* genomic data by alignment and searching the *EsDscam* cDNA sequence. The preliminary *EsDscam* gene was improved by PCR to avoid sequencing errors and gaps.

### Analysis of Epitope I and Epitope II

The amino-terminal halves of Ig2 and Ig3 were encoded by variable alternative exons represented by DNA stretches of approximately 195 bp for Ig2 (exon 4) and 169 bp for Ig3 (exon 6) in *E. sinensis*. Based on the structural characteristics in *D. melanogaster* (21) and the similarities in the secondary structure of Dscam among *D. melanogaster, D. magna* (23), and *E. sinensis*, the epitope I and epitope II sequences of *EsDscam* were identified using PSIPRED^1^ (24). Both exon 4 and exon 6 in *Es*Dscam contribute to each epitope in such a manner that their N-terminal segments encode epitope I and the C-terminal segments encode epitope II. In exon cluster 4, the 12 amino acids between the conserved 7Q and the 18V were considered to belong to epitope I, and the sequence of approximately 18 amino acids after 43W were considered to belong to epitope II (23). In exon cluster 6, the eight amino acids after 15R were considered to belong to epitope I, and the eight amino acids before the conserved LLC motif were considered to belong to epitope II (23) (Figure S3 in Supplementary Material). For analysis of the conservation of different epitope sequences, the identified epitope I and epitope II sequence logos of exon 4 and exon 6 were generated using WebLogo.^2^

### Hemocyte Culture

Primary culture of *E. sinensis* hemocyte was performed according to established techniques (25). Briefly, hemolymph was collected from the non-sclerotized membrane of the posterior walking leg using a 10-ml sterile syringe preloaded with 5-ml pre-cooled sterile anticoagulant (0.14 M NaCl; 0.1 M glucose; 30 mM trisodium citrate; 26 mM citric acid; 10 mM EDTA, pH 4.6) at a ratio of 1:1 (25). The collected hemolymph was immediately centrifuged at 300 × *g* for 10 min at 4°C, and then the serum was removed and washed with PBS. The isolated hemocytes were gently resuspended in Leibovitz’s L-15 medium (Sigma-Aldrich, USA) supplemented with 1% antibiotics [10,000 U/ml penicillin, 10,000 µg/ml streptomycin (Gibco, USA)], 0.2 mM NaCl (676 ± 5.22 mOsm/kg), pH 7.20–7.40, and subsequently were counted using an automated cell counter (Invitrogen Countess) before seeding 4 ml (1 × 10^5^ cells/ml) in 60-mm dishes (Corning Inc., USA).

### Bacterial Challenge and Sample Collection

Bacterial strains [*V. parahemolyticus* (BYK00036), *S. aureus* (BYK0113), *Bacillus subtilis* (BYK0123), and *Aeromonas hydrophila* (BYK00335)], which are main pathogenic bacteria that caused heavy diseases in aquaculture (26), were gifted by the National Pathogen Collection Center for Aquatic Animals (Shanghai Ocean University, Shanghai, China). Bacteria were cultured overnight in LB medium and collected by centrifugation at 5,000 × *g* for 5 min, then washed three times, and resuspended in sterile PBS. The colony-forming unit (CFU) counts of bacteria were determined by plating the diluted suspension onto agar plates.

For *in vitro* bacterial stimulation, experiments were performed according to previously reported methods (25). Briefly, overnight cultures of bacteria were heat inactivated at 72°C for 20 min and collected by centrifugation. Heat-inactivated *V. parahemolyticus, S. aureus, B. subtilis*, and *A. hydrophila* (1 × 10^8^ microbes per milliliter, 50 µl) were added separately to one 60-mm dish (Corning) with about 4 × 10^5^ of hemocytes. Sterile PBS (50 µl) was used as the control. After 0, 2, 4, 6, 12, 24, 36, and 48 h of bacterial stimulation, hemocytes and hemocyte culture medium were both collected and stored at −80°C. The collected hemocytes samples were used to extract the total RNA for *Es*Dscam mRNA expression analyses, the hemocyte culture medium samples were used for *Es*Dscam protein expression analyses. Three independent repeats were performed with at least three crab for each sample.

For *in vivo* bacterial infection, *S. aureus* and *V. parahemolyticus* were both cultured overnight in LB broth at 37°C and adjusted to 1 × 10^8^ CFU per milliliter, after that, mixed pathogen (2 × 10^7^ CFU, 200 µl) were injected into hemolymph from the non-sclerotized membrane of the posterior walking leg, then different tissues that including intestine, heart, brain, hepatopancreas, gill, testis, stomach, cell-free hemolymph, and hemocytes were collected at 24 h post-challenge and stored at −80°C. The collected samples were used to extract the total protein for *Es*Dscam protein expression analyses, three independent repeats were performed with three crab for each sample.

### Recombinant Expression, Purification, and Antiserum Production

To produce antiserum against *Es*Dscam, a fusion protein was expressed in *E. coli* according to a previously reported method (27). A PCR fragment representing the extracellular domains of *Es*Dscam from FNIII3 to FNIII6 was amplified using specific primers (*Es*Dscam-anti-F and *Es*Dscam-anti-R; Table 1). Subsequently, the PCR products were purified and digested with restriction enzymes (*EcoR*I and *Xho*I) for ligation of the final DNA fragment into the pET-28a (+) vector (Novagen, USA). The recombinant plasmid pET-28a (+)-*Es*Dscam was transformed into *E. coli* Rosetta (DE3) cells (Transgen, China) for expression of the recombinant *Es*Dscam protein. Fusion protein expression was induced by exposure to four different sets of conditions: 1 mM isopropyl-β-d-thiogalactoside (IPTG) for 3 h at 37°C; 0.25 mM IPTG for 3 h at 37°C; 1 mM IPTG for 3 h at 30°C; and 0.25 mM IPTG for 3 h at 30°C. The fusion protein was purified with Ni-NTA resin (Transgen, China) according to the manufacturer’s instructions. Subsequently, rabbit antiserum against *Es*Dscam was prepared according to a standard procedure (28), and the antiserum titer was determined by ELISA. Three recombinant proteins [r*Es*Dscam_**(4.6,6.9)**_, r*Es*Dscam_**(4.24,6.19)**_, and r*Es*Dscam_**(4.12,6.20)**_] containing Ig1–Ig4 and another three proteins containing Ig1–Ig2, Ig2–Ig3, and Ig3–Ig4 were also expressed in *E. coli* with the pET-28a (+) vector after induction with IPTG as described above. The specific primers are listed in Table 1. Subsequently, rabbit antisera against r*Es*Dscam_**(4.24,6.19)**_ and r*Es*Dscam_**(4.12,6.20)**_ were prepared using the same method for detection of the specific protein isoforms expressed by the bacteria. Furthermore, to confirm the interaction of *Es*-sDscam and *Es*-mDscam by far-Western assay, the myc-tag recombinant proteins [r*Es*Dscam_**(4.24,6.19)**_ and r*Es*Dscam_**(4.12,6.20)**_] were expressed in *E. coli* with the pBAD-myc-His vector after induction with l-arabinose. For use *in vivo*, these proteins received an additional wash with excess 0.1% Triton X-114 at 4°C before the final elution was performed to remove contaminating endotoxins according to a previously reported method (29). The endotoxin content of the proteins was detected using a lipopolysaccharide (LPS) detection kit (GenScript, China) according to the manufacturer’s instructions.

**Table 1:** Oligonucleotide primers used in this study.

Primers	Sequence (5′–3′)
**Real-time PCR**	
*Es*Dscam-RT-F	CAATGTGAGGGTAACTGATGATGGC
*Es*Dscam-RT-R	TACTGAATCTTGAACTGAGGTGGAG
*Es*Dscam-TM-F	CGTTTCCTGGCTGCCTGACTGGTGG
*Es*Dscam-TM-R	CACAGCCACACAGACGACAACAATG
*Es*Dscam-exon4-F	GCCTCCAACCCCCACGGCTCAGTCC
*Es*Dscam-exon4-R	CGACACTTGTAACTCTTGAAGCCAT
*Es*Dscam-exon6-F	CCGCCTCACCCAGGAAACCCGTCTC
*Es*Dscam-exon6-R	CAGTCTCCACACTCTCGCCGCCCAC
*Es*Dscam-exon4.24-F	CAGACGTACGTGACGCGGGTG
*Es*Dscam-exon4.24-R	CATGTTCTCGGCCCGAAGTG
*Es*Dscam-exon6.20-F	GTGCCGCGACGTGCGCAGTTC
*Es*Dscam-exon6.20-R	CTGAAGGCCGGGACAGGGTAGG
β-actin-RT-F	GCGAGAAATCGTGCGAGACAT
β-actin-RT-R	CCGAGGAAGGAAGGCTGGAAGAG
**Protein expression**	
*Es*Dscam-anti-F	GGAATTCCCCCAGCAGCCCCCTCAGGATGTG *EcoR*I
*Es*Dscam-anti-R	CCGCTCGAG**TTA**GGCTCCAGTAAGAGTAAGAGTTGC *Xho*I
*Es*Dscam_4.6,6.9_-F	CGGGATCCGACTTCAGCAACTCG *BamH*I
*Es*Dscam_4.6,6.9_-R	CCCAAGCTT**TTA**CTCCTGGTCATTCCTCACG *Hind*III
*Es*Dscam_4.24,6.19_-F	CGGGATCCGGCCCTGTCATCGTGGAAG *BamH*I
*Es*Dscam_4.24,6.19_-R	CCCAAGCTT**TTA**CTGAGCAGACTCCTGGTCATTCC *Hind*III
*Es*Dscam_4.24,6.20_-F	CGGGATCCCCGGACAACCGCGTGGAC *BamH*I
*Es*Dscam_4.24,6.20_-R	CCCAAGCTT**TTA**CTGGGGTGGCTCAAAGCGTCCT *Hind*III
*Es*DscamIg1-2-F	CGGGATCCGGCCCTGTCATCGTGGAAG *BamH*I
*Es*DscamIg1-2-R	CCCAAGCTT**TTA**GTCGGAGATGACGAGGCGTCC *Hind*III
*Es*DscamIg2-3-F	CGGGATCCCGAGCTGTCCACGTGCGC *BamH*I
*Es*DscamIg2-3-R	CCCAAGCTT**TTA**CAGCACAGTCTCCACACTCTCG *Hind*III
*Es*DscamIg3-4-F	CGGGATCCATCTCCGATGCGCTCTCCAGT *BamH*I
*Es*DscamIg3-4-R	CCCAAGCTT**TTA**CTGAGCAGACTCCTGGTCATTCCT *Hind*III
*Es*Dscam_4.24,6.19_-pBAD-F	CCGCTCGAGAGGCCCTGTCATCGTGGAAG *Xho*I
*Es*Dscam_4.24,6.19_-pBAD-R	CGGAATTCCGCTGAGCAGACTCCTGGTCATTCC *EcoR*I
*Es*Dscam_4.24,6.20_-pBAD-F	CCGCTCGAGACCGGACAACCGCGTGGAC *Xho*I
*Es*Dscam_4.24,6.20_-pBAD-R	CGGAATTCCGCTGGGGTGGCTCAAAGCGTCCT *EcoR*I
*Es*Dscam_4.24,6.19_-pAc-His-F	GGAATTC**ATG**GGCCCTGTCATCGTGGAAG *EcoR*I
*Es*Dscam_4.24,6.19_-pAc-His-R	ACCGGTCTGAGCAGACTCCTGGTCATTCC *Age*I
*Es*Dscam_4.24,6.19_-pAc-V5-F1	GGAATTC**ATG**GGCCCTGTCATCGTGGAAG *EcoR*I
*Es*Dscam_4.24,6.19_-pAc-V5-R1	AGGGTTAGGGATAGGCTTACCCTGAGCAGACTCCTGGTC
*Es*Dscam_4.24,6.19_-pAc-V5-R2	CGTAGAATCGAGACCGAGGAGAGGGTTAGGGATAGGC
*Es*Dscam_4.24,6.19_-pAc-V5-R3	CCGCTCGAG**TTA**CGTAGAATCGAGACCGAGGAG *Xho*I
*Es*Dscam_4.24,6.20_-pAc-V5-F1	GGAATTCCCGGACAACCGCGTGGAC *EcoR*I
*Es*Dscam_4.24,6.20_-pAc-V5-R1	AGGGTTAGGGATAGGCTTACCCTGGGGTGGCTCAAAGCGTCCT
*Es*Dscam_4.24,6.20_-pAc-V5-R2	CGTAGAATCGAGACCGAGGAGAGGGTTAGGGATAGGC
*Es*Dscam_4.24,6.20_-pAc-V5-R3	CCGCTCGAG**TTA**CGTAGAATCGAGACCGAGGAG *Xho*I
**RNA interference**	
dsGFP-F	GCGTAATACGACTCACTATAGAGTGCTTCAGCCGCTACCC
dsGFP-R	GCGTAATACGACTCACTATAGGCGCTTCTCGTTGGGGTC
ds*Es*Dscam-TM-F	GCGTAATACGACTCACTATAGTGAATGTGCAACTCTTACTCTTACT
ds*Es*Dscam-TM-F	GCGTAATACGACTCACTATAGTCACAGCCACACAGACGACAACAAT
si*Es*Dscam-exon4.24-F	CAGACGUACGUGACGCGGGUG
si*Es*Dscam-exon4.24-R	CACCCGCGUCACGUACGUCUG
si*Es*Dscam-exon6.20-F	GUGCCGCGACGUGCGCAGUUC
si*Es*Dscam-exon6.20-R	GAACUGCGCACGUCGCGGCAC
siGFP-F	AGUGCUUCAGCCGCUACCC
siGFP-R	GGGUAGCGGCUGAAGCACU

*EcoRI, XhoI, BamHI, HindIII, and AgeI restriction sites are marked by single underlining; the double underlining indicates the V5 tag sequence; the start codon and stop codons are shown in bold text*.

### Expression Profiles of *Es*Dscam

The total RNA from bacteria-stimulated hemocytes was extracted using TRIzol Reagent (Invitrogen) according to the manufacturer’s instructions. Total RNA (1 µg) was then reverse transcribed using the PrimeScript™ RT reagent kit with gDNA Eraser (Perfect Real-Time) (TaKaRa, Japan) and the synthesized first-strand cDNA was used as a template for RT-qPCR. RT-qPCR was performed using SYBR Premix Ex Taq (Tli RNaseH Plus) (TaKaRa) and the CFX96™ Real-Time System (BioRad, USA). The *EsDscam*-RT-F and *EsDscam*-RT-R primers (Table 1) were used to analyze the expression patterns of the *EsDscam* gene in hemocytes after 0, 2, 4, 6, 12, 24, 36, and 48 h of bacterial stimulation. β-actin gene was used as the internal reference. All RT-qPCRs were completed in triplicate and normalized to the control gene. The PCR conditions were 95°C for 30 s, followed by 40 cycles of 95°C for 5 s and 66°C for 30 s. The melting curves from 60 to 95°C were then determined. *EsDscam* expression levels were calculated using the 2^−ΔΔCT^ (ΔΔCT = ^ΔCT^*EsDscam*-^ΔCT^β-actin) method (30). Three independent experiments were performed, and the results present the mean ± SD.

The *Es*Dscam protein expression profiles after bacterial challenge *in vivo* and *in vitro* were both determined by Western blotting. Briefly, for detecting total *Es*Dscam protein expression profiles, the protein samples from different tissues were extracted using the Minute™ series of protein extraction kits (Invent, USA) and separated by 6% SDS-PAGE, the non-stimulation crabs’ protein samples were also extracted and analyzed by Western blotting. Proteins (50 µg) were then transferred to a PVDF membrane, and blocked with 5% non-fat milk in PBST for 1 h at room temperature. After blocking, the membrane was incubated with primary antiserum against *Es*Dscam (1:1,000) overnight at 4°C, followed by incubation with goat anti-rat IgG H&L (Alexa Fluor® 790) (1:2000; Abcam, UK). After several washing steps with PBST, the target band was visualized using the eECL Western Blot kit (Cwbiotech, China) and imaged with ChemiDoc XRS (BioRad, USA). The expression of β-actin (Transgen, China) was detected as the reference. To detect the soluble *Es*Dscam expression profiles, the hemocyte culture medium (10 µl) was separated by 6% SDS-PAGE, and analyzed by Western blotting as described above.

### Exon 4 and 6 Variants in *Es*Dscam

To detect the alternatively spliced exon 4 and exon 6 isoforms of *EsDscam* after bacterial stimulation, total RNA was extracted from the hemocytes at a specific time point post-stimulation with *S. aureus* (6 h), *B. subtilis* (48 h), *A. hydrophila* (12 h), and *V. parahemolyticus* (6 h); a non-bacteria-stimulated (0 h) group served as the control. According to previously reported methods (18, 27, 31), the hemocyte from three independent culture dishes were used to prepare each sample, and the exon 4 and exon 6 isoforms of *Es*Dscam were amplified by RT-PCR using the *Es*Dscam exon4-F/R and *Es*Dscam exon6-F/R primers for TA cloning. Individual colonies (*n* = 80) containing the exon 4 and exon 6 fragments, respectively, were selected randomly from each agar plate and sequenced using the universal primer. The sequencing results for the clones from the five different groups were aligned and analyzed using ClustalX 2.0. The observed number of exon 4 and exon 6 isoforms in each group was calculated by Excel. Two independent experiments were performed, and the results represent the mean colonies.

### Peptide Synthesis

Based on the results of exon variants of *EsDscam* detected after bacterial stimulation as well as the conservation sites of epitope II sequences, 11 peptides (10 representing epitope II of exon 4 and exon 6 and 1 from the non-alternative splicing region of *Es*Dscam gene representing a conserved motif as a control) were synthesized by GL Biochem (Shanghai, China) and dissolved in ddH_2_O at a final concentration of 50 µg/ml.

### Bacteria-Binding Assays

The assay of bacteria binding with peptide was detected according to a previously reported method (27). Briefly, 10 ml of overnight cultures of *S. aureus, B. subtilis, A. hydrophila*, and *V. parahemolyticus* were collected and adjusted to 1 × 10^9^ CFU/ml. The bacteria were centrifuged at 5,000 × *g* for 5 min, washed three times, resuspended in sterile PBS, and then heat inactivated at 72°C for 20 min. For the binding assay, each peptide (5 µg, 100 µl) or control was mixed with 100 µl bacterial suspension (1.0 × 10^8^ microbes). The mixture was then incubated at 28°C for 1 h under gentle agitation. After centrifugation 5,000 × *g* for 5 min and washing with PBS, the samples were centrifuged again under the same conditions, and the supernatant was removed. The bacteria pellet was resuspended in 100 µl, 0.1 M carbonate/bicarbonate buffer (pH 9.6), and used to coat a 96-well plate by incubation for 2 h at 37°C. Subsequently, the wells were blocked with blocking buffer (2% BSA in PBST) for 2 h at room temperature and then incubated with goat anti-His mouse monoclonal antibody (1:1,000) (Transgen, China) overnight at 4°C. Plates were washed with PBST before incubation with goat anti-mouse HRP-conjugated secondary antibody (1:5,000) (Transgen, China) for 1 h. To detect the binding signal, freshly prepared TMB substrate (Sigma) was added to the plates for 10 min, and the reaction was terminated by the addition of stop solution (2 M HCl). Subsequently, the absorbance of each well was measured within 30 min at a wavelength of 450 nm using a microplate reader (Thermo Scientific, USA).

The assay of bacteria binding with recombinant protein was detected according to previously reported method (16). Briefly, the purified recombinant proteins [r*Es*Dscam_**(4.6,6.9)**_, r*Es*Dscam_**(4.24,6.19)**_, and r*Es*Dscam_**(4.12,6.20)**_] (5 µg) were mixed with 1 × 10^8^ microbes of three strains of bacteria (*S. aureus, V. parahemolyticus*, and *A. hydrophila*). The mixture was then incubated by gentle agitation at 28°C for 1 h. The bacteria pellets were pre-washed three times with 1 ml PBS buffer (pH 8.0) and isolated by centrifugation at 5,000 × *g* for 5 min, then resuspended in 100 µl PBS. Finally, the samples were subjected to Western blotting using the anti-His mouse monoclonal antibody (1:1,000; Transgen, China). Bacteria incubated in the absence of the recombinant proteins were used as controls. ELISAs were also used to quantify the microorganism binding activities of r*Es*Dscams as described above. The dissociation constants (*K*_d_) and the maximum binding (*B*_max_) parameters were calculated using the GraphPad Prism version 5.0 software. Three independent ELISA experiments were performed, and the results represent the mean ± SD.

### Bacteria Clearance Assays

Bacteria clearance assays were performed according to a previously reported method (32). Bacterial suspensions (*S. aureus* and *V. parahemolyticus*) were cultured overnight in LB broth at 37°C and adjusted to OD_600_ = 0.2. The recombinant proteins (r*Es*Dscam_**4.6,6.9**_, r*Es*Dscam_**4.24,6.19**_, and r*Es*Dscam_**4.12,6.20**_) (5 µg) were then mixed with 100 µl of the suspensions (1 × 10^8^ CFU) and incubated under gentle agitation at 28°C for 1 h. After thorough washing with PBS, the bacteria were collected by centrifugation at 5,000 × *g* for 5 min and resuspended in 200 µl of PBS, then injected into each crab as described earlier, and the hemolymph was collected at 30 min post-injection. After serial dilution, the number of residual bacteria was determined by plating the samples onto LB agar plates. Three to five crabs were used in each group, and each experiment was repeated three times. The results are presented as the mean ± SD. Bacteria incubated in the absence of recombinant proteins were used as controls.

### Fluorescent Labeling of Bacteria and Phagocytosis Assay

Phagocytosis assays (*in vitro* and *in vivo*) were performed according to previously reported methods (16, 32–34). Overnight-cultured *S. aureus* and *V. parahemolyticus* were heat killed and fluorescein isothiocyanate (Sigma, USA) conjugated. The bacterial suspension (1 × 10^8^ microbes per milliliter, 1 ml) in PBS was mixed and incubated with 500 µg of recombinant proteins by gentle rotation for 1 h at 28°C to ensure full coating. The bacteria were pelleted and washed three times with PBS by centrifugation.

For the *in vitro* analysis, cultured hemocytes (4 × 10^5^ cells) were stimulated firstly with heat-killed bacteria (1 × 10^6^ microbes) for 12 h and washed with PBS twice before the addition of approximately 1 × 10^6^ (40 µl) FITC-conjugated microbes coated with recombinant proteins. After incubation for 1 h at room temperature, the cells were washed twice with PBS and stained with trypan blue (2 mg/ml) for 20 min to quench the non-phagocytosed bacteria. The cells were then washed three times with PBS, and stained with DAPI for 1 min. Subsequently, phagocytosis was observed using a fluorescence microscope (Olympus BX71, Japan). Five hundred hemocytes were counted for each sample, which was from three crab. The hemocytes (500 cells) were counted and the phagocytic rate was determined as the ratio of the number of phagocytic cells containing fluorescent bacteria to the total number of cells.

For the *in vivo* analysis, the bacterial suspension (100 µl) was injected into the hemolymph of crab from the one of the posterior walking legs. After 1 h, the hemocytes (6 × 10^5^ cells) were isolated from the other one of the posterior walking legs which was the different wound-sites and centrifuged at 300 × *g* for 10 min at 4°C, followed by washed with PBS three times. Subsequently, the phagocytosis rate in 1 ml of each sample was determined by flow cytometry using a CytoFLEX flow cytometry (Beckman, USA), and the data were analyzed using CytExpert software. Ten thousand hemocytes were counted for each sample, which was from three crab. The experiments were repeated three times.

### RNA Interference

For knockdown of *EsDscam* expression, a partial *Es*Dscam non-alternatively spliced cDNA fragment was amplified by PCR with primers linked to the T7 promoter (Table 1) and used as the template to produce dsRNA with an *in vitro* T7 Transcription Kit (Fermentas, Burlington, ON, Canada). The control GFP dsRNA was synthesized in the same way with primers listed in Table 1. To test whether the expression of *Es*Dscam could be suppressed, the dsRNA was then dissolved in RNase-free water and transfected into *E. sinensis* primary cultured hemocytes using Lipofectamine 3000 (Thermo, USA) at a final concentration of 5 nM. At least three crab were used for testing of RNAi efficiency at 24 h post dsRNA transfection. To detect the effect of *Es*Dscam knockdown on phagocytosis, dsRNA pre-treated hemocytes were then subjected to bacteria stimulation for a further 12 h.

For knockdown of exon 4.24- and exon 6.20-containing *Es*Dscam isoforms expression, siRNA methods were used to silence the expression of exon 4.24- and exon 6.20-containing *Es*Dscam isoforms due to the short epitope II sequences. Briefly, siRNA against exon 4.24- and exon 6.20-containing *Es*Dscam isoforms and GFP (as control) were synthesized by GenePharma (Shanghai, China); the primer sequences used are listed in Table 1. These siRNAs were used to transfect *E. sinensis* primary hemocytes using the siRNA-Mate reagent (GenePharma, China) according to the manufacturer’s instructions. At least three crab were used for testing of RNAi efficiency at 24 h post siRNA transfection.

After validating that targeted gene expression could be silenced by the dsRNA and siRNA, crab hemocyte were used for RNAi, which divided into four parts. The first part was hemocyte transfected each with 5 nM dsRNA, the second part was hemocyte transfected each with 5 nM siRNA, and the last two parts were hemocyte each for GFP dsRNA and GFP siRNA transfection as control. After bacteria pre-stimulation, hemocyte were stimulated by bacteria coated with recombinant proteins for another 1 h and phagocytosis were analyzed as described earlier. Results were analyzed by Student’s *t*-test and expressed as the mean ± SD from three independent experiments.

### Immunocytochemistry

Hemocyte immunocytochemistry was performed according to a previously reported method (34). Briefly, the cultured primary hemocytes (approximately 4 × 10^5^ cells) were seeded onto a 24 mm × 24 mm glass cover slips in 35-mm dishes. After RNAi-mediated knockdown of *Es*Dscam expression or stimulation with bacteria, the cells were washed twice with PBS and fixed immediately with 4% paraformaldehyde in sterile PBS for 15 min. The hemocytes were washed again with PBS and exposed to 0.5% Triton X-100 for 10 min. After washing three times with PBS, the hemocytes were blocked with 3% BSA in PBS for 2 h and incubated with polyclonal rabbit antiserum against *Es*Dscam (1:200) and anti-His mouse monoclonal antibody (1:1,000; Transgen, China) overnight at 4°C. After washing with PBST for removal of unbound antibody, cells were stained with goat anti-rat IgG H&L (FITC Conjugated) (1:100; CWBIO, China) and goat anti-mouse IgG H (Rhodamine Red-X Conjugated) (1:100; CWBIO, China) for 2 h at 37°C. After washing with PBST, cells were stained with DAPI for 1 min, and the cover slips were removed for observation under a fluorescence microscope (Olympus BX71, Japan).

### Far-Western Blotting and Co-immunoprecipitation Assays

The interactions between the same and different isoforms of *Es*Dscam *in vitro* were evaluated using the far-Western blotting assays performed according to previously described methods with slight modifications (35, 36). Briefly, r*Es*Dscam_**4.24,6.19**_-His (50 µg) was separated by 12% SDS-PAGE and transferred to a nitrocellulose membrane. The membrane containing the bait protein was then washed with denaturation buffer [6 M guanidine–HCl in basic buffer (10% glycerol, 100 mM NaCl, 1 mM EDTA, 1 mM DTT, 1% Tween-20, 20 mM Tris, pH 7.5)] at room temperature for 30 min with gentle agitation. The membrane was then washed sequentially in 3 M guanidine–HCl for 30 min at room temperature, in 1 M guanidine–HCl for 30 min at 4°C, and in 0.1 M guanidine–HCl for 30 min at 4°C. Finally, the membrane was washed in basic buffer for 30 min and blocked with the blocking buffer (2% non-fat milk in basic buffer) for 2 h. Subsequently, the crab plasma protein (1 mg), His peptide (1 mg), r*Es*Dscam_**4.24,6.19**_-myc (1 mg), or r*Es*Dscam_**4.12,6.20**_-myc (1 mg) was added to the interaction buffer (2% milk in basic buffer) at 4°C overnight. The non-interacting proteins were then removed by washing in PBST, and the bound proteins were detected with anti-*Es*Dscam (1:500), anti-His (1:1,000; Transgen, China), and anti-Myc (1:1,000; Transgen, China) antibodies by Western blotting.

No crab or crustacean cell lines are currently available; however, the *Drosophila* S2 cell line has been widely used to study the function of crustacean genes (37–39). To confirm the interaction between the same and different isoforms of Dscam *in vivo*, S2 cells were cultured for coimmunoprecipitation assays according to previously reported methods (39). Briefly, *Drosophila* S2 cells were seeded in 24-well plates and cultured at 28°C overnight in Schneider’s insect medium (Sigma) supplemented with 10% FBS (Life Technologies, USA). After 24 h, cells were cotransfected with pAc5.1-V5-His A, pAc5.1-r*Es*Dscam_**4.12,6.20**_-V5 or pAc5.1-r*Es*Dscam_**4.24,6.19**_-His, and pAc5.1-r*Es*Dscam_**4.24,6.19**_-V5 using Lipofectamine^®^ 3000 transfection reagent (Thermo, USA) according to the manufacturer’s instructions. pAc5.1-V5-His A and pAc5.1-r*Es*Dscam_**4.12,6.20**_-V5 were used as controls. At 36 h post-transfection, S2 cells were washed twice with PBS before proteins were extracted using RIPA buffer followed by centrifugation at 14,000 × *g* for 10 min. The supernatant containing a pool of r*Es*Dscam_**4.24,6.19**_-His and r*Es*Dscam_**4.24,6.19**_-V5 proteins was pre-cleared with 30 µl of Protein A beads at 4°C for 40 min with agitation. Subsequently, the mixture was centrifuged at 12,000 × *g* for 10 min to remove the beads. The supernatant was then incubated with 10 µg His and V5 antibodies overnight at 4°C under gentle rotation. Protein A beads were then added to the mixture and incubated for 1 h at 4°C to capture the antibodies. The beads were collected by centrifugation and then washed with PBS and resuspended in the SDS-PAGE loading buffer for separation by SDS-PAGE and Western blot analysis using the His or V5 antibody.

## Results

### Genome Organization and Alternatively Spliced Exons

**Figure 1:**
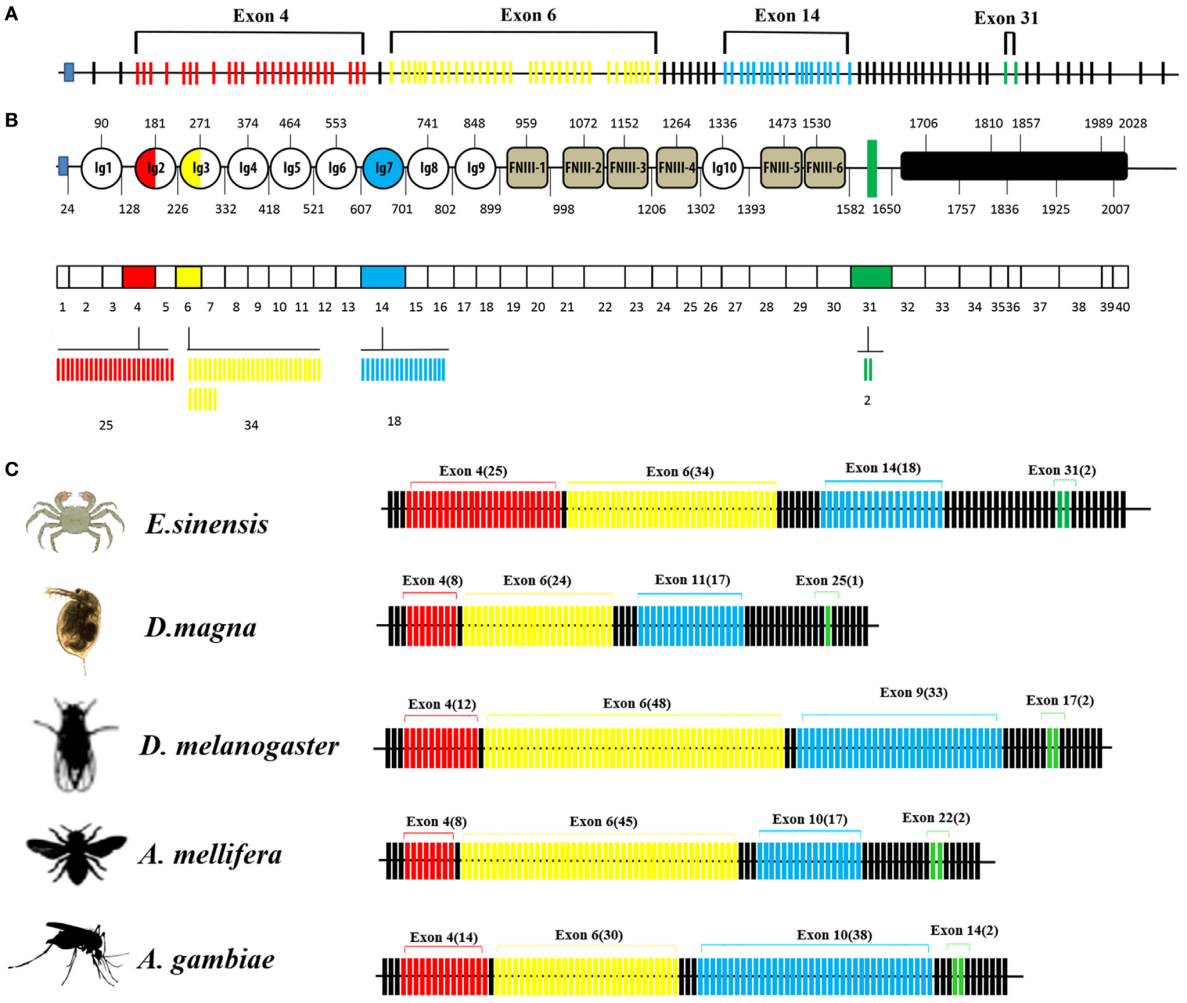
Genome structure of Dscam gene. **(A)** *Eriocheir sinensis* Dscam genomic DNA structure. *Es*Dscam mRNA comprises 40 exons (short lines); 36 constant exons are indicated by black lines and 4 hypervariable exons are indicated by colored lines. Exon 4 contains 25 variants (red), exon 6 contains 34 variants (yellow), exon 14 contains 18 variants (blue), and exon 31 contains 2 variants (green). **(B)** *Es*Dscam protein domain and mRNA structure. (*Upper panel*) The alternatively spliced exons encode the N-terminal half of Ig2 (red semicircle) and Ig3 (yellow semicircle), all of Ig7 (blue circle), and the transmembrane domain (green rectangle); (*middle panel*) constant exons are shown as white boxes and the mutually exclusive alternative splicing exons are shown in the same color scheme as that used for the protein structure. *Es*Dscam mRNAs contain only one of each of the alternative splicing exons; (*lower panel*) the variant numbers of mutually exclusive alternative splicing exons are indicated by colored lines using the same color scheme as that used for the protein structure. **(C)** Comparison of *Dscam* gene within arthropod species. The number of exons is shown. Black lines represent constant exons. The hypervariable exons in the exon 4, 6, 9, and 17 clusters of *Drosophila melanogaster* are shown in red, yellow, blue, and green, respectively. The number of variable exons within each array in each species is displayed above each array. The exon 10 arrays of *Anopholes gambiae* and *A. mellifera*, the exon 11 array of *D. magna* and the exon 14 array of *Eriocheir sinensis* correspond to the exon 9 array of *Drosophila*. Similarly, the exon 14 and 22 arrays of *A. gambiae* and *A. mellifera*, the exon 25 array of *D. magna* and the exon 31 array of *E. sinensis* correspond to the exon 17 array of *Drosophila*.

The organization of the *Dscam* gene in arthropods usually comprises clusters of variable exons flanked by constant exons (14, 40). The *EsDscam* gene (GenBank accession number: KT175608) (Figure 1A) contains 114 exons, of which 40 account for the mature mRNA (Figure 1B, middle panel) encoding the protein structure that shares high similarity with *D. melanogaster* Dscam (Figures S1A,B in Supplementary Material). The alternative splicing regions are located in sequences encoding the extracellular and transmembrane regions (Figure 1B, upper panel). Similar to other arthropod species (14, 23, 41, 42), the extracellular region of *Es*Dscam has three hypervariable sites: the exon 4 cluster that encodes part of the Ig2 domain and has 25 variants, the exon 6 cluster that encodes part of the Ig3 domain and has 34 variants, and the exon 14 cluster that encodes the entire Ig7 domain and has 18 variants (Figure 1B, lower panel). In addition, there are two variants of the transmembrane domain (TM) region (Figure 1B, lower panel). The variable number of spliced exons in arthropod species confers the potential for expression of different isoforms, and even the lowest number maintained a high degree of divergence of 10^4^ (Figure 1C). Taken together, these findings demonstrated that *Es*Dscam forms the domain organization of the 9(Ig)-4(FNIII)-Ig-2(FNIII)-TM-cytoplasmic tail, with the possible generation of 30,600 isoforms *via* alternative splicing of three Ig domain regions (Ig2, Ig3, and Ig7) and a TM.

### *Es*Dscam Is Upregulated in Hemocytes Post-Bacterial Stimulation

**Figure 2:**
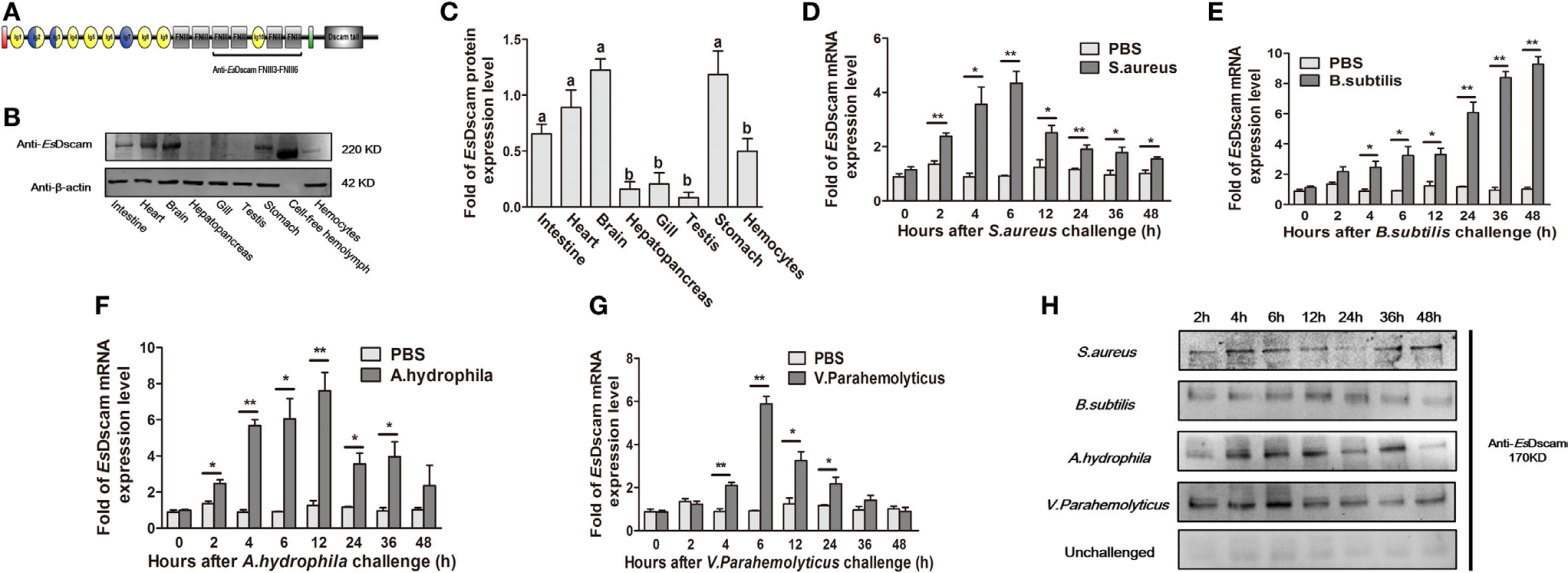
Identification of *Es*Dscam as a bacteria-sensitive gene in crab hemocyte. **(A)** Schematic diagram of the conserved *Es*Dscam FNIII3–FNIII6 region recognized by the *Es*Dscam Ab. **(B,C)** Tissue distribution of *Es*Dscam. (*Left panel*) The protein expression was analyzed by Western blot with β-actin detected as the reference. Each sample was from four crab. (*Right panel*) ImageJ was used to scan the Western blot bands from three independent repeats. Relative expression level of *Es*Dscam/β-actin are shown, and the value are expressed as relative arbitrary units. Results were analyzed by one-way ANOVA and the letters (a, b) presented significant differences (*p* < 0.05). **(D–G)** Expression profile of *Es*Dscam mRNA after bacterial stimulation in the hemocytes. RNA was extracted at each time point from experimental and control crab. qRT-PCR was performed to examine the expression of *Es*Dscam in each sample with *β-actin* as the reference gene. The expression was normalized to that in PBS stimulated crab. Three independent repeats were performed with at least three crab for each sample, and results are expressed as the mean ± SD. Data were analyzed by Student’s *t*-test. *p < 0.05, **p < 0.01. **(H)** Expression profile of soluble *Es*Dscam protein in hemocyte culture medium after bacterial stimulation. The protein expression was analyzed by Western blot with unchallenged hemocyte as the control. Data are representative of two independent repeats.

*Es*Dscam protein expression was analyzed by Western blotting using anti-*Es*Dscam serum (Figure S2 in Supplementary Material) generated using the purified extracellular region of FNIII3 to FNIII6 (Figure 2A), and with preimmune serum as negative controls. The results showed that non-specific signals from the antibodies were not detected, so it is acceptable for use in subsequent studies. *Es*Dscam expression was detected in hemocytes, intestine, heart, brain, stomach, and cell-free hemolymph after pathogen stimulation (Figures 2B,C). The expression of Dscam in hemocytes has been widely confirmed in arthropod species (13, 18, 20, 27, 31, 43, 44), its expression in *E. sinensis* hemocytes post-pathogen stimulation remains to be established, although it has been tested in *E. sinensis* in relation to PAMPs (Glu, LPS, and PG) stimulation (43). In this study, qRT-PCR analysis of the temporal expression patterns of *EsDscam* in hemocytes after bacterial stimulation showed that *EsDscam* was significantly upregulated and peaked at 6 h (approximately fourfold upregulation) post-stimulation with *S. aureus* (Figure 2D), at 48 h (approximately eightfold upregulation) post-stimulation with *B. subtilis* (Figure 2E), at 12 h (approximately eightfold upregulation) post-stimulation with *A. hydrophila* (Figure 2F), and at 6 h (approximately sevenfold upregulation) post-stimulation with *V. parahemolyticus* (Figure 2G). However, no suitable primers were available to distinguish soluble Dscam from membrane-bound Dscam since the two forms express the same extracellular region (13), therefore we were unable to analyze the expression profile of soluble *EsDscam* using this strategy. To address this issue, the expression profile of soluble *Es*Dscam protein in the cell-free hemolymph post-bacterial stimulation was analyzed by Western blotting. The results revealed marked upregulation of *Es*Dscam post-stimulation with *S. aureus, B. subtilis, A. hydrophila*, and *V. parahemolyticus* (Figure 2H), indicating that soluble *Es*Dscam may play a role in antimicrobial responses.

### Dscam Alternative Splicing in Response to Infection

**Figure 3:**
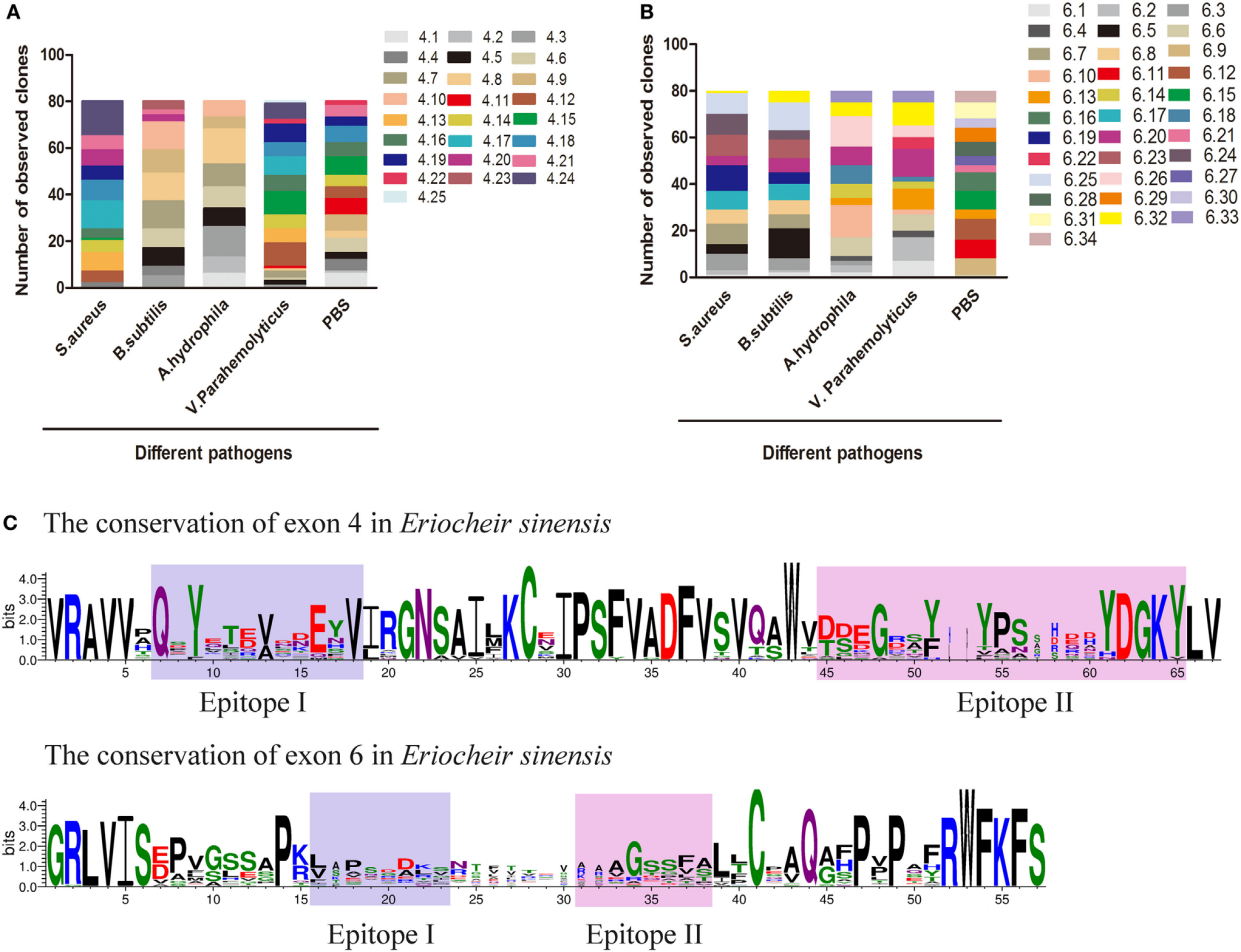
Preferential variants of exon 4 and exon 6 after bacterial stimulation in hemocyte. **(A)** Number of observed clones of exon 4 in hemocytes post-challenged with various bacteria and PBS (control). The variants of exon 4 were analyzed by cloning and sequencing 80 clones from each group. Individual colonies were randomly selected from each sample. All assays were repeated on two independent occasions, and values represent the mean number of observed clones from two independent experiments. The serial number of exon 4/6 was assigned and the amino-acid sequences of exon 4/6 are shown in Figure S3 in Supplementary Material. **(B)** Number of observed clones of exon 6 post-challenge with various bacteria and PBS. **(C)** Differential sequence conservation of epitopes I and II of *Es*Dscam following alternative splicing of exon 4 and exon 6. (*Upper panel*) Sequence logo representation of the conservation of exon 4 variants in *Eriocheir sinensis*; (*lower panel*) sequence logo representation of the conservation of exon 6 variants in *E. sinensis*. Bits (*y*-axis) indicate units of evolutionary conservation.

A global assessment of Dscam alternative splicing in *D. melanogaster* using custom-made oligo-arrays demonstrated that almost all alternative exon 4 and exon 6 sequences were expressed in fat bodies and hemocytes, while only a limited subset of exon 9 sequences were expressed in these cell types (13). Furthermore, challenge of the hemocyte-like immune-competent cell line, Sua5B, with bacteria, fungi, and pathogen-associated surface molecules resulted in rapid and specific changes in exon usage in an acute phase-responsive manner (16). To investigate the alternative splicing pattern of *EsDscam* in hemocytes post-bacterial stimulation, two gene specific primer pairs (Table 1) were designed to amplify alternatively spliced Ig2 and Ig3 regions. Bacteria were found to induce obvious variation in *EsDscam* splice forms, while stimulation with PBS resulted in the predominant production of splice forms containing exon 4.15 (Figure 3A) and exon 6.12 (Figure 3B). Stimulation with *S. aureus* resulted in the predominant production of splice forms containing exon 4.24 (Figure 3A) and exon 6.19 (Figure 3B). Stimulation with *B. subtilis* resulted in the predominant production of splice forms containing exon 4.7/4.8/4.9/4.10 (Figure 3A) and exon 6.5 (Figure 3B). Stimulation with *A. hydrophila* resulted in the predominant production of splice forms containing exon 4.8 (Figure 3A) and exon 6.10 (Figure 3B). Stimulation with *V. parahemolyticus* resulted in the predominant production of splice forms containing exon 4.12/4.15 (Figure 3A) and exon 6.20 (Figure 3B). These result suggested that the expression profile of Dscam isoforms may varied upon different bacteria stimulation, and the predominant exons may provide us some clues for constructing recombinant proteins that specific binding with the original bacteria. The partition of exon 4 and exon 6 epitopes was assigned based on the crystal structure of *D. melanogaster* Dscam (21) and the secondary structure of Dscam (Figure S1C in Supplementary Material). For comparisons of the similarities and phylogenetics among the epitopes, we analyzed the sequence conservation of epitopes I and II of exons 4 and 6 in *E. sinensis* (Figure 3C), which showed low conservation for both epitopes I and II. These results were similar with the previous report (21) that within a species that epitope I is low conserved, while across species that epitope I is comparative highly conserved. However, the potential role of epitope II mediated non-Dscam ligands binding (21) has not been empirically tested so far.

### *Es*Dscam-Specific Binding With Bacteria

**Figure 4:**
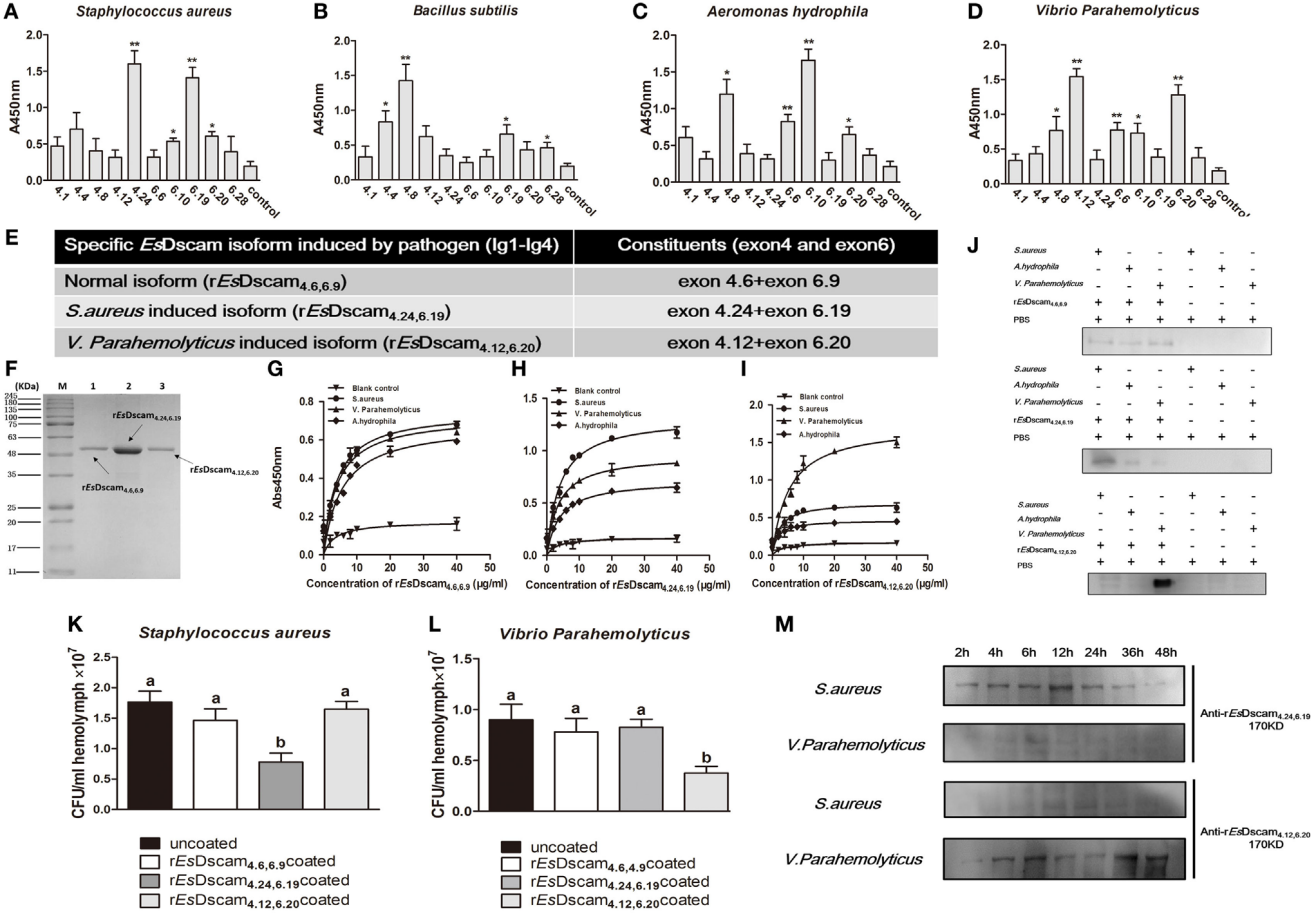
Soluble *Es*Dscam-specific binding with bacteria and promotes its clearance. **(A–D)** Bacterial binding specificity of epitope II in exon 4/exon 6. The binding activity between selected peptides and different bacteria were analyzed by ELISA with peptides that containing the conserved motif as control. Three independent repeats were performed, and results are expressed as the mean ± SD. Data were analyzed by Student’s *t*-test. *p < 0.05, **p < 0.01. **(E)** Specific recombinant *Es*Dscam isoforms induced by pathogens. Normal isoform composed of alternative spliced exon 4.6, alternative spliced exon 6.9, and constant exon designated r*Es*Dscam**_4.6,6.9_**; *Staphylococcus aureus*-induced isoform composed of alternative spliced exon 4.24, alternative spliced exon 6.19, and constant exons designated r*Es*Dscam**_4.24,6.19_**; *Vibrio parahemolyticus*-induced isoform composed of alternative spliced exon 4.12, alternative spliced exon 6.20, and constant exons designated r*Es*Dscam**_4.12,6.20_**. **(F)** Purified recombinant specific *Es*Dscam isoform. Three recombinant r*Es*Dscam protein were expressed from the pET-28a (+) vector in *Escherichia coli* Rosetta (DE) cells and purified by affinity chromatography, followed by SDS-PAGE detection. **(G–I)** Quantitative binding of *Es*Dscam with bacteria. Microtiter plates were coated with heat-inactivated bacteria which were pre-coated with serial concentrations of proteins and air dried at 37°C for 2 h. After the wells were blocked with 2% BSA, the bound proteins were then detected by ELISA using an anti-His-tag Ab. Data represent the mean ± SD of three independent experiments. **(J)** Microorganism binding assays of r*Es*Dscam proteins. The coated samples of bacteria with or without r*Es*Dscam protein and PBS were boiled for 10 min and separated by 12% SDS-PAGE for Western blotting using an anti-His-tag antibody, with PBS incubated with microorganisms as control. Data are representative of two independent repeats. **(K,L)** *Es*Dscam promotes bacteria clearance in crab. Each r*Es*Dscam (5 µg) was incubated with 100 µl of the *S. aureus* (*left panel*) or *V. parahemolyticus* (*right panel*) suspension (OD_600_ = 0.2) for 1 h. The bacteria were washed and suspended in 200 µl of PBS. Then, 200 µl of the suspension was injected into the crab, and hemolymph was collected 30 min later. The number of residual bacteria in the hemolymph was determined by plating onto LB agar plates. Uncoated bacteria were used as the control. Three to five crabs were used for each group. Three independent repeats were performed, and results are expressed as the mean ± SD. Data were analyzed by one-way ANOVA and the letters (a, b) presented significant differences (*p* < 0.05). **(M)** Specific *Es*Dscam was highly induced after bacteria stimulation in cell-free hemolymph. Protein from cell-free hemolymph was extracted at each time point after bacterial stimulation and analyzed by Western blot using anti-r*Es*Dscam**_4.24,6.19_** and anti-r*Es*Dscam**_4.12,6.20_** antibodies. Each sample was from four crab. Data are representative of two independent repeats.

Alternative splicing allows Dscam to produce a broad repertoire of receptors, thereby increasing the probability of recognizing and defending against a broad spectrum of pathogens (45). To confirm the existence of bacteria-specific *Es*Dscam isoforms, we first analyzed the epitope II sequences in every alternatively spliced exon of the Ig2 and Ig3 regions, and classified into five or six groups based on the similarity of these sequences (Figure S1E in Supplementary Material). Subsequently, we synthesized 11 peptides based on the epitope II sequence divergence and the predominant production of splice forms detected following bacterial stimulation. The synthesized 11 peptides contains 5 peptides from epitope II from the exon 4, 5 peptides from epitope II from the exon 6, and control peptide from the non-alternative splicing region of *Es*Dscam gene (Figure S1D in Supplementary Material). Exons 4.24 and 6.19 were implicated in strong binding activity with *S. aureus* (Figure 4A), exon 4.8, with *B. subtilis* (Figure 4B), exons 4.8 and 6.10, with *A. hydrophila* (Figure 4C), and exons 4.12 and 6.20, with *V. parahemolyticus* (Figure 4D). These results indicated that exon 4.24 and 6.19 involved *Es*Dscam isoform may specific binding with *S. aureus*, exon 4.12 and 6.20 involved *Es*Dscam isoform may specific binding with *V. parahemolyticus*. However, since it is difficult to synthesis all epitope II sequences in every alternative spliced exon, we cannot draw the whole picture of bacteria-specific binding exons at this time, and we think epitope II from some untested exons might also specific binding with particular bacteria.

To test the ability of *Es*Dscam_**(4.24, 6.19)**_ and *Es*Dscam_**(4.12, 6.20)**_ binding specifically with its inducing bacteria, we used a prokaryotic expression system to generate recombinant proteins, with r*Es*Dscam_**(4.6, 6.9)**_ protein as control (Figures 4E,F; Figure S4 in Supplementary Material). ELISA and Western blot analyses showed that r*Es*Dscam_**(4.6, 6.9)**_ bound all the bacterial strains tested with low affinity (Figures 4G,J, upper panel), while r*Es*Dscam_**(4.24, 6.19)**_ bound *S. aureus* with higher affinity than either *A. hydrophila* or *V. parahemolyticus* (Figures 4H,J, middle panel), and r*Es*Dscam_**(4.12, 6.20)**_ bound only *V. parahemolyticus* with high affinity (Figures 4I,J, lower panel). Furthermore, *in vivo* bacteria clearance assays in *E. sinensis* showed that *S. aureus* was only cleared efficiently when pre-coated with r*Es*Dscam_**(4.24, 6.19)**_ (Figure 4K), while *V. parahemolyticus* was only cleared efficiently when pre-coated with r*Es*Dscam_**(4.12, 6.20)**_ (Figure 4L). These results revealed the specific binding activity of bacteria-induced *Es*Dscam splice forms only with the original inducing bacteria; however, significant induction of these specific *Es*Dscam isoforms in cell-free hemolymph post-bacterial infection remained to be confirmed. To address this issue, we detected the levels of *Es*Dscam_**(4.24, 6.19)**_ and *Es*Dscam_**(4.12, 6.20)**_ proteins in hemolymph at different time-points post-infection by Western blot analysis. *Es*Dscam_**(4.24, 6.19)**_ expression was found to be induced significantly by *S. aureus* rather than *V. parahemolyticus*, while soluble *Es*Dscam_**(4.12, 6.20)**_ was induced significantly by *V. parahemolyticus* but not by *S. aureus* (Figure 4M), which implies the role of soluble *Es*Dscam in immune defense.

### Bacteria-Specific Binding by *Es*-sDscam Promotes Phagocytosis

**Figure 5:**
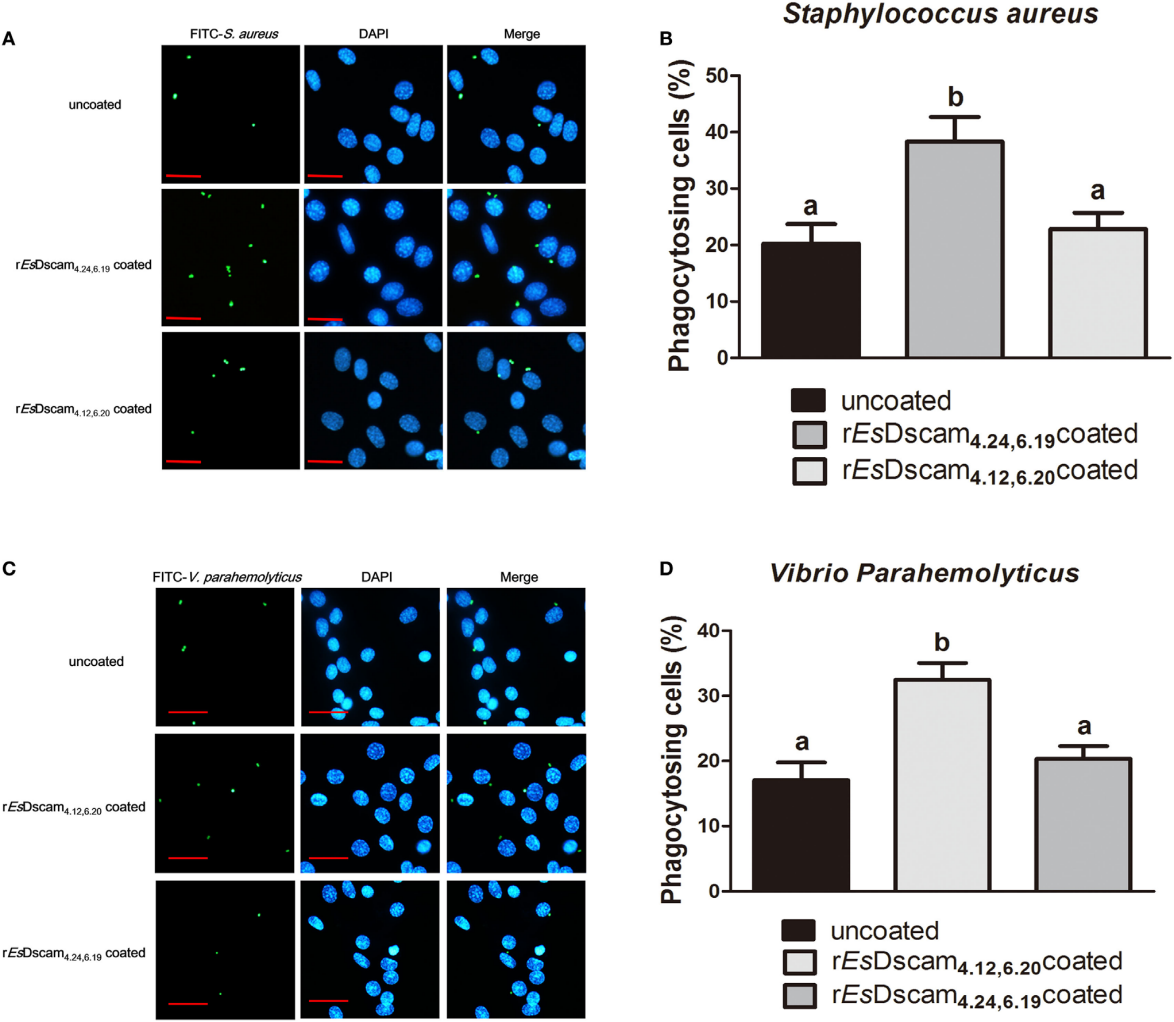

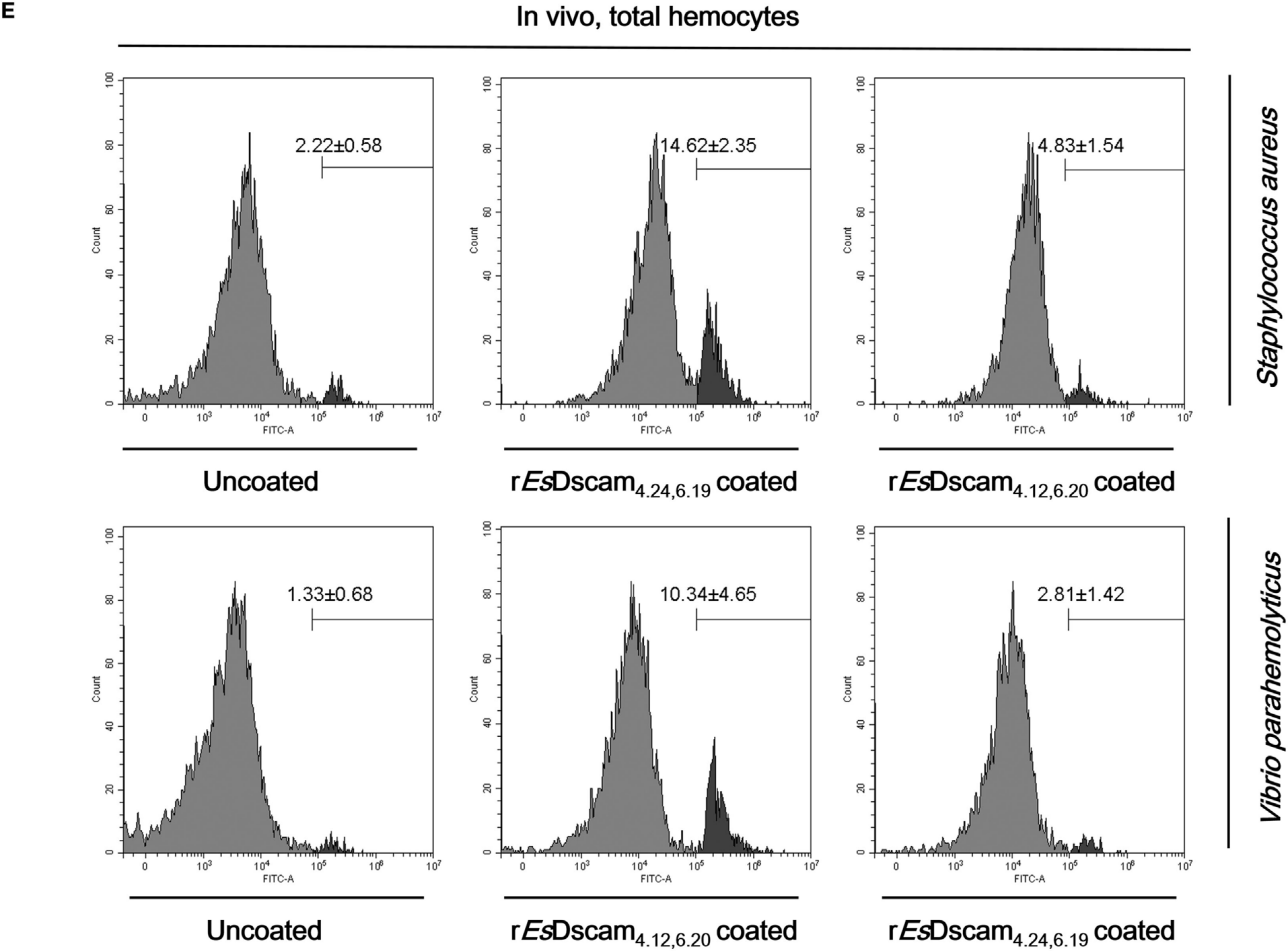
Soluble *Es*Dscam enhanced the phagocytosis of bacteria in hemocytes. **(A,B)** r*Es*Dscam**_4.24,6.19_** promote phagocytosis of *Staphylococcus aureus in vitro*. *S. aureus* (1 × 10^6^ microbes) labeled with FITC and then coated with r*Es*Dscam**_4.24,6.19_** or r*Es*Dscam**_4.12,6.20_** protein was used to stimulate cultured hemocyte, with uncoated bacteria as control. Hemocytes was then collected 1 h later, washed with PBS. After staining with DAPI and quenching with trypan blue, hemocytes were observed under a fluorescence microscope. Scale bar = 15 μm. The phagocytosis (*left panel*) were detected by fluorescence microscopy, data representative three independent repeats. The phagocytosis rate (*right panel*) were calculated according to the formula presented in Section “Materials and Methods” based on counting 500 hemocytes in each experiment. Three independent repeats were performed, and results are expressed as the mean ± SD. Data were analyzed by one-way ANOVA and the letters (a, b) presented significant differences (*p* < 0.05). **(C,D)** r*Es*Dscam**_4.12,6.20_** promote phagocytosis of *Vibrio parahemolyticus in vitro*. The same bacteria concentration, as well as the protocol and statistical method were used as described earlier. **(E)** *Es*Dscam promote phagocytosis of bacteria *in vivo*. *S. aureus* and *V. parahemolyticus* were heat-inactivated and labeled with FITC before coating with r*Es*Dscam**_4.24,6.19_** and r*Es*Dscam**_4.12,6.20_**, respectively. The bacteria were then injected into the hemolymph of crab and hemocytes were collected 1 h later for flow cytometric analysis. A total 10,000 hemocytes were counted for each sample. Three independent repeats were performed, and results are expressed as the mean ± SD.

Dscam-regulated phagocytosis has been confirmed in some other arthropod species (13, 16, 46); therefore, we investigated the ability of *Es*-sDscam to reduce the number of bacteria *via* phagocytosis regulation and the function of bacteria-specific binding in this process. For this purpose, the hemocytes were pre-stimulated with the bacteria for 12 h, and we pre-coated different strains of FITC-labeled bacteria with r*Es*Dscam_(4.24, 6.19)_ and r*Es*Dscam_(4.12, 6.20)_, then performed the phagocytosis assay *in vitro*. *In vitro* phagocytosis assays using different strains of FITC-labeled bacteria showed that r*Es*Dscam_**(4.24, 6.19)**_ enhanced the rate of *S. aureus* phagocytosis by approximately 100% (Figures 5A,B) and r*Es*Dscam_**(4.12, 6.20)**_ enhanced the rate of *V. parahemolyticus* phagocytosis by approximately 70% (Figures 5C,D). By contrast, r*Es*Dscam_**(4.24, 6.19)**_ and r*Es*Dscam_**(4.12, 6.20)**_ had no significant effect on the rate of *V. parahemolyticus* and *S. aureus* phagocytosis (Figures 5A–D). These results were confirmed *in vivo* by flow cytometry to differentiate hemocytes from bacteria and cell debris (Figure 5E). The results also showed that bacteria-specific binding by *Es-*sDscam promoted a marked increase in phagocytosis of the original inducing bacteria, while only a marginal effect on the non-specific binding bacteria was observed (Figure 5E). These results demonstrated an essential role of bacteria-specific binding in *E*s-sDscam-promoted phagocytosis.

### Truncated *Es*-sDscam Abolished the Promotion of Phagocytosis

**Figure 6:**
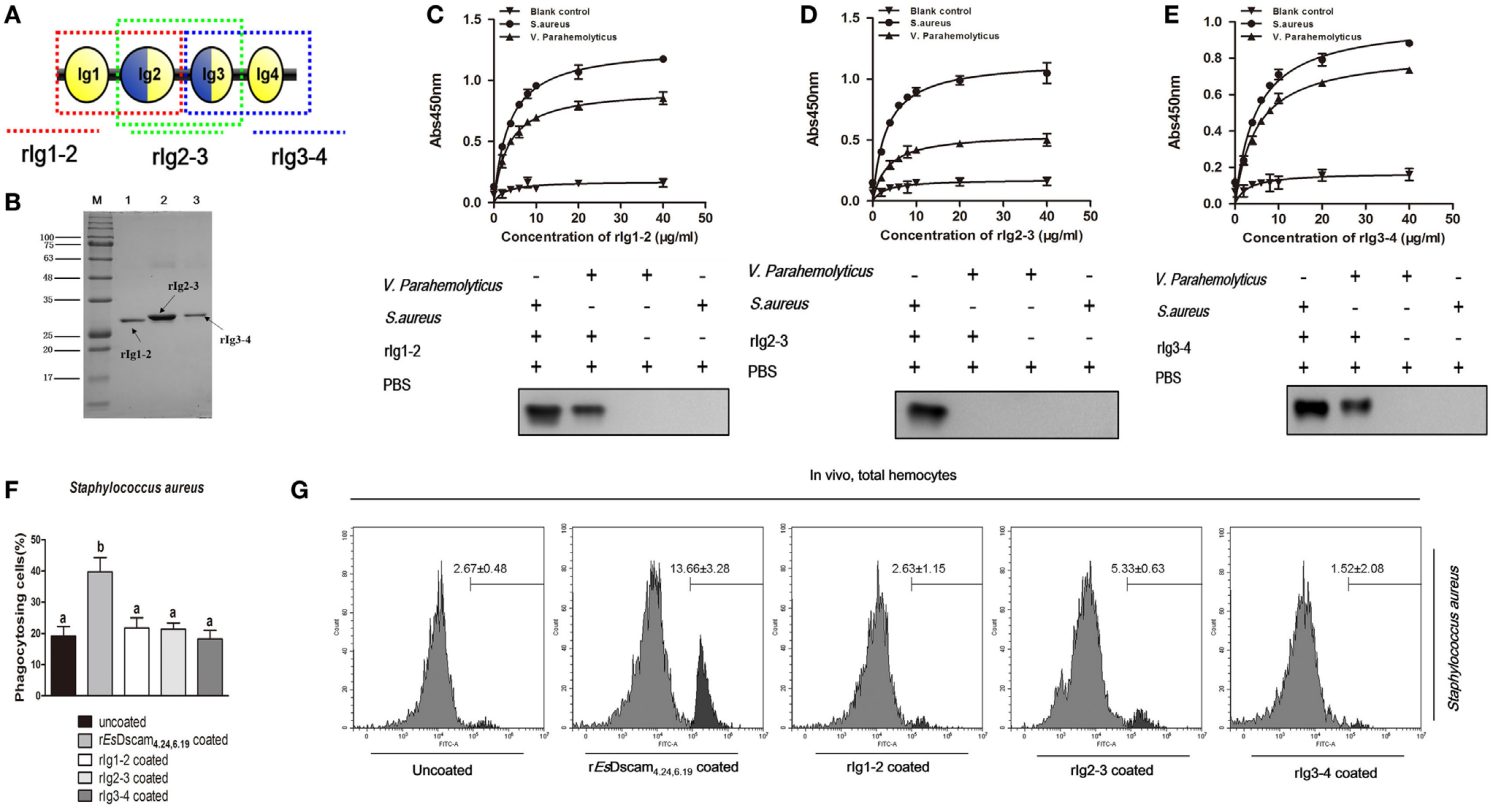
Truncated soluble *Es*Dscam exhibit variation in bacteria-binding ability and loss of the capacity to promote phagocytosis. **(A)** Schematic diagram of truncated r*Es*Dscam**_4.24,6.19_**. The red, green, and blue dotted lines represents the rIg1–2, rIg2–3, and rIg3–4 proteins, respectively. The three domains were expressed *in vitro* using a prokaryotic expression system (results of the construction, expression, and purification of the recombinant isoforms are shown in Figure S4 in Supplementary Material). **(B)** Recombinant truncated soluble *Es*Dscam proteins were analyzed by SDS-PAGE. Three proteins were expressed from the pET-28a (+) vector in *Escherichia coli* Rosetta (DE) cells and purified by affinity chromatography. **(C–E)** Quantitative binding of truncated r*Es*Dscam proteins with bacteria. The binding activity between truncated r*Es*Dscam proteins and bacteria were detected by ELISA using anti-His-tag antibodies. PBS incubated with microorganisms was used as the control. Absorbance data (*upper panel*) represent the mean ± SD of three independent experiments. Western data (*lower panel*) are representative of two independent repeats. **(F)** Truncated *Es*Dscam has slight effect on phagocytosis of bacteria *in vitro*. Each recombinant protein was incubated with FITC-labeled *Staphylococcus aureus* and then used to stimulate hemocyte, with r*Es*Dscam_4.24,6.19_ as positive control. Three independent repeats were performed, and results are expressed as the mean ± SD. Data were analyzed by one-way ANOVA and the letters (a, b) presented significant differences (*p* < 0.05). **(G)** Truncated *Es*Dscam has slight effect on phagocytosis of bacteria *in vivo*. *S. aureus* was heat-inactivated and labeled with FITC before coating with each r*Es*Dscam protein. The bacteria were then injected into the hemolymph of crab and hemocytes were collected 1 h later for flow cytometry analysis, with uncoated bacteria as control. A total 10,000 hemocytes were counted for each sample. Three independent repeats were performed, and results are expressed as the mean ± SD.

X-ray crystallography of the structure of the amino-terminal four Ig domains (Ig1–Ig4) of two distinct Dscam isoforms in *D. melanogaster* revealed a horseshoe configuration (21). Since Ig1 and Ig4 are constant domains, this configuration should be a general feature of the structure of all Dscam isoforms (21). The 3D-structure of the amino-terminal four Ig domains (Ig1-Ig4) of r*Es*Dscam isoform also revealed a horseshoe configuration (Figure S1C in Supplementary Material), which was very similar to one Dscam isoform in *D*. *melanogaster*. Therefore, we hypothesized that the complete Ig1–Ig4 structure may play a critical role in bacteria-specific binding and the subsequent regulation of phagocytosis. To test that hypothesis, we used a prokaryotic expression system to generate three truncated recombinant *E*s-sDscam proteins (Ig1–Ig2, Ig2–Ig3, and Ig3–Ig4) based on r*Es*Dscam_**(4.24, 6.19)**_ (Figures 6A,B; Figure S4 in Supplementary Material). Evaluation of bacterial binding by ELISA and Western blotting demonstrated that these truncated r*Es*Dscam proteins retained the ability to bind bacteria, albeit with impaired specificity (Figures 6C,D). Briefly, the truncated recombinant proteins had almost no effect on phagocytosis compared with negative control (Figure 6F). To confirm these results *in vivo*, flow cytometry was conducted to analyze the phagocytosing cells among the total hemocytes. The results obtained were in accordance with the *in vitro* study data showing that truncated *Es*-sDscam has only a slight effect on the promotion of phagocytosis compared with that induced by r*Es*Dscam_**(4.24, 6.19)**_ (Figure 6G). Collective results demonstrated the role of the complete *Es*Dscam Ig1-Ig4 structure in the promotion of phagocytosis, while the truncated recombinant proteins had almost no effect on phagocytosis, and prompted us to explore the possible mechanism underlying this phenomenon.

### Membrane-Bound *Es*Dscam Regulated the Promotion of Phagocytosis by Soluble *Es*Dscam

**Figure 7:**
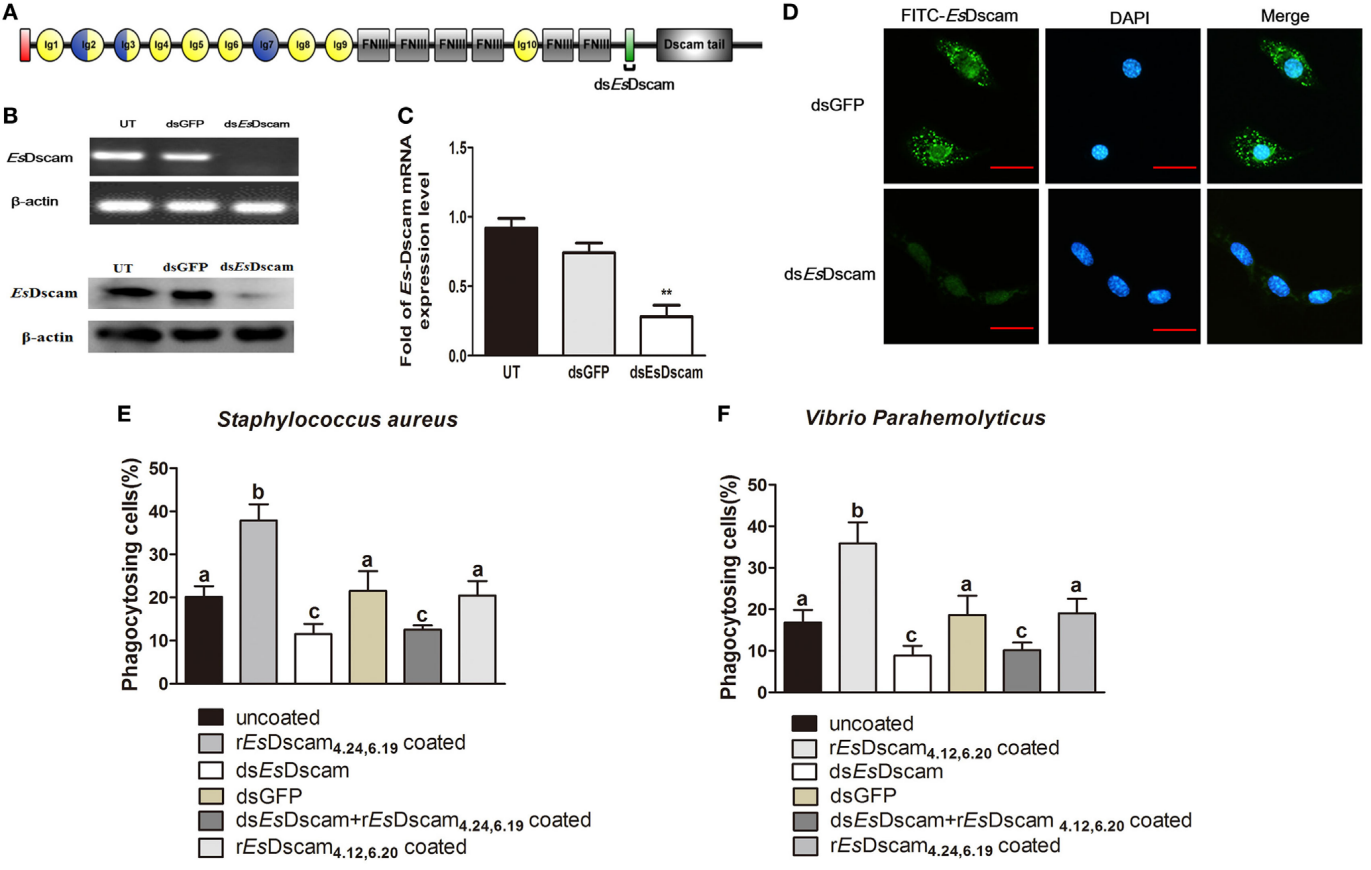
Soluble *Es*Dscam promotes phagocytosis *via* membrane-bound *Es*Dscam in hemocytes. **(A)** Schematic diagram showing the RNAi region recognized by primers in the conserved transmembrane region of ds*Es*Dscam. **(B–D)** Knockdown of *Es*Dscam expression. Crab hemnocyte were transfected with *Es*Dscam dsRNA (5 nM) and equal amounts of GFP dsRNA served as a control. RNA samples were collected at 24 h post-transfection to check the silencing efficiency by RT-PCR (*left panel*) and qRT-PCR (*right panel*) with β-actin as a reference. And the protein samples also were collected and detected by Western blotting with β-actin as a reference. RT-PCR data are representative of two independent repeats. At least three crab were used for each sample, and qRT-PCR were performed three times. The results are expressed as the mean ± SD, data were analyzed by Student’s *t*-test. **p < 0.01. (*Right panel*) Immunocytochemical analysis of *Es*Dscam dsRNA transfected hemocyte showing decreased *Es*Dscam protein expression compared with dsGFP-treated control cells. Scale bar = 15 µm. Data are representative of three independent repeats. **(E,F)** Membrane-bound *Es*Dscam regulate soluble *Es*Dscam promoted phagocytosis in hemocyte. Rate of hemocyte phagocytosis of **(E)** *Staphylococcus aureus* and **(F)** *Vibrio parahemolyticus* following *Es*Dscam knockdown. Phagocytic rates of hemocytes were calculated according to the formula presented in the Materials and Methods section. Three independent repeats were performed, and results are expressed as the mean ± SD. Data were analyzed by one-way ANOVA and the letters (a, b, c) presented significant differences (*p* < 0.05).

Phagocytosis, which is an essential process performed by unicellular organisms and many cell types found in metazoans, begins with the engagement of phagocytic receptors that activate numerous signaling pathways (46). In mammals, antibody-bound (opsonized) pathogens are recognized by Fc receptors (46); however, in arthropods, it is unclear whether soluble Dscam-opsonized bacteria are recognized by a dedicated receptor or through homotypic interactions with membrane-bound Dscam. To test the function of membrane-bound *Es*Dscam as a phagocytic receptor in soluble *Es*Dscam-regulated phagocytosis, we used dsRNA targeting the transmembrane region of *Es*-mDscam (Figure 7A) to knockdown expression to approximately 10% of the level detected in the control (Figures 7B,C). The efficiency of the RNAi-mediated reduction in expression was also confirmed by immunohistochemical analysis of *Es*-mDscam protein expression (Figure 7D). Using this approach, we showed that *Es*-mDscam knockdown caused a significant reduction in the rate of phagocytosis in *S. aureus*- and *V. parahemolyticus*-stimulated hemocytes (Figures 7E,F). More importantly, *Es*-mDscam knockdown significantly inhibited *Es*Dscam_**(4.24, 6.19)**_-induced *S. aureus* phagocytosis (Figure 7E) and *Es*Dscam_**(4.12, 6.20)**_-induced *V. parahemolyticus* phagocytosis (Figure 7F). These observations demonstrated the role of membrane-bound *Es*Dscam in soluble *Es*Dscam-regulated phagocytosis. In addition, hemocyte phagocytosis was reduced by knockdown of *Es*-mDscam or culture with r*Es*Dscam_**(4.24, 6.19)**_. Although these results suggest the participation of *Es*-mDscam in *Es*-sDscam-regulated phagocytosis, whether soluble Dscam-opsonized bacteria are recognized through membrane-bound Dscam remains to be clarified.

### Membrane-Bound *Es*Dscam Acts as a Phagocytic Receptor for Soluble *Es*Dscam

**Figure 8:**
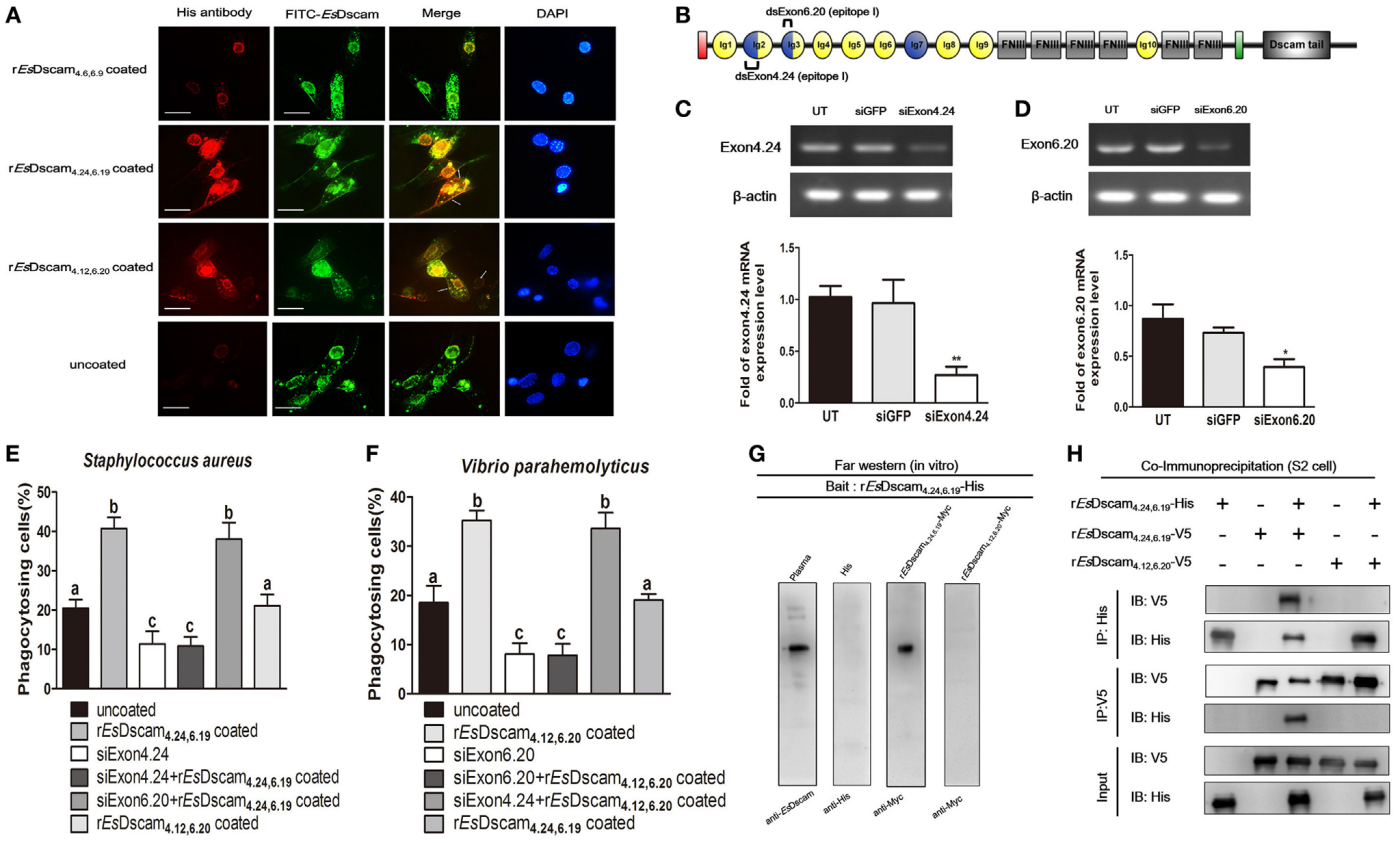
Interaction of membrane-bound and soluble *Es*Dscam is essential for promotion of phagocytosis. **(A)** Colocalization of *Es*-mDscam and *Es*-sDscam in hemocyte surface. Each group that including r*Es*Dscam**_4.6,6.9_**, *Staphylococcus aureus* coated r*Es*Dscam**_4.24,6.19_**, and *Vibrio parahemolyticus* coated r*Es*Dscam**_4.12,6.20_** was used to stimulate cultured crab hemocyte. Hemocytes were collected 1 h later and subjected to immunocytochemistry detected by the antibodies of *Es*Dscam (membrane-bound *Es*Dscam) and His-tag (soluble *Es*Dscam). Scale bar = 15 µm. Data are representative of two independent repeats. **(B)** Schematic diagram showing the RNAi region recognized by siExon 4.24 and siExon 6.20 primers in the epitope I region. **(C,D)** Knockdown of exon 4.24 and exon 6.20 expression. Crab hemocyte were transfected with 5 nM exon 4.24 siRNA (*left panel*) or exon 6.20 siRNA (*right panel*), with equal amounts of GFP siRNA served as a control. RNA samples were collected at 24 h post-transfection to check the silencing efficiency by RT-PCR with β-actin as a reference. RT-PCR data are representative of two independent repeats. At least three crab were used for each sample, and qRT-PCR were performed three times. The results are expressed as the mean ± SD, data were analyzed by Student’s *t*-test. **p < 0.01, *p < 0.05. **(E,F)** Specific membrane-bound *Es*Dscam regulate soluble *Es*Dscam promoted phagocytosis in hemocyte. Rate of hemocyte phagocytosis of **(E)** *Staphylococcus aureus* and **(F)** *V. parahemolyticus* following knockdown of exon 4.24 and exon 6.20, respectively. Phagocytic rates of hemocytes were calculated according to the formula presented in Section “Materials and Methods.” At least 15 fields were included and data represent the mean ± SD of three independent experiments. The results were analyzed statistically using one-way ANOVA and the letters (a, b, c) presented significant differences (*p* < 0.05). **(G)** Interaction between membrane-bound and soluble *Es*Dscam *in vitro*. The bait protein r*Es*Dscam**_4.24,6.19_**-His was first separated by SDS-PAGE, transferred onto a nitrocellulose membrane, and renatured by adding a series of different concentration of guanidine-HCl. The undetected proteins were added to recognize the bait protein on the membrane before the attached proteins were detected by Western blotting using the specific antibodies. Data are representative of two independent repeats. **(H)** Interaction between membrane-bound and soluble *Es*Dscam *in vivo*. Lysates from S2 cells transiently transfected with pAc5.1-r*Es*Dscam**_4.24,6.19_-**His, together with pAc5.1-r*Es*Dscam**_4.12,6.20_-**V5 or pAc5.1-r*Es*Dscam**_4.24,6.19_-**V5, were subjected to immunoprecipitation with anti-His Ab or anti-V5 Ab, followed by Western blot analysis with anti-His Ab and anti-V5 Ab. The input controls were also analyzed by Western blot. Data are representative of two independent repeats.

To evaluate the possible interaction between soluble and membrane-bound forms of *Es*Dscam, colocalization of *Es*-sDscam and *Es*-mDscam in hemocytes was investigated by immunohistochemistry. *Es*-sDscam was found to colocalize with *Es*-mDscam following coculture with its specific binding bacteria (Figure 8A). Since homotypic interactions may be the basis for the binding of mDscam with sDscam, we speculated that only *Es*-mDscam with the same alternative spliced exons was able to enhance *Es*-sDscam-regulated phagocytosis. To test this hypothesis, siRNA targeting exons 4.24 and 6.20 (Figure 8B) were transfected into hemocytes leading to significant silencing of exon 4.24 involved membrane-bound *Es*Dscam (exon 4.24-*Es*Dscam) that corresponding to soluble r*Es*Dscam_**(4.24, 6.19)**_ (Figure 8C) and exon significant silencing of exon 6.20 involved membrane-bound *Es*Dscam (exon 6.20-*Es*Dscam) that corresponding to soluble r*Es*Dscam_**(4.12, 6.20)**_ (Figure 8D). Knockdown of the both exons led to significantly reduced phagocytosis (Figures 8E,F). Interestingly, knockdown of exon 4.24-*Es*Dscam significantly reduced r*Es*Dscam_**(4.24, 6.19)**_-enhanced phagocytosis, while knockdown of exon 6.20-*Es*Dscam did not (Figure 8E). Furthermore, knockdown of exon 6.20-*Es*Dscam significantly reduced r*Es*Dscam_**(4.12, 6.20)**_-enhanced phagocytosis, while knockdown of exon 4.24-*Es*Dscam did not (Figure 8F). The interaction of *Es*-sDscam and *Es*-mDscam was confirmed *in vitro* and *in vivo* by analysis of myc-tagged forms of the r*Es*Dscam_**(4.24,6.19)**_ and r*Es*Dscam_**(4.12,6.20)**_ proteins using far-Western blot (Figure 8G) and coimmunoprecipitation (Figure 8H), which revealed that *Es*-sDscam and *Es*-mDscam interactions occur only between the same isoforms. These results suggest that *Es*-mDscam functions as a phagocytic receptor for *Es*-sDscam *via* homotypic interactions.

## Discussion

A vast repertoire of immune receptors is required to provide adaptive immune defense against an equally vast and rapidly evolving pool of potential pathogens. Antigen receptor diversity in mammals is generated by somatic gene rearrangement and increased in B cells by somatic hyper-mutation. By contrast, these processes are absent in arthropods (46) and the mechanisms responsible for generation of the required receptor repertoire in these species remain to be elucidated. Interestingly, the recent discovery of the role of Dscam in host defense in insects (13, 15, 16) and shrimp (19, 44) has provided important insights.

The extreme diversity of the proteins encoded by *Dscam* genes is almost unique (47) and despite the consistency of multiple variants within the three exon clusters across all crustaceans, these variants are not conserved across species (19). In species other than *D. melanogaster*, the numbering of orthologous exon clusters varies due to differences in the positions of exon–exon boundaries (16). Mutually exclusive alternative splicing of Dscam pre-mRNA ensures that each mRNA contains only one of the possible variants from each of the three alternative exon clusters (14, 48). Across species, these alternatively spliced exons encode the N-terminal regions of the Ig2 and Ig3 domains and the entire Ig7 domain. These domains are located in the extracellular portion of the protein, with the potential to produce more than 10,000 splice forms from a single gene. However, there are major differences in types of alternative splicing from chelicerates to pancrustaceans, even within pancrustaceans there could be room for differences in the mode of expression resulting in modulations of Dscam role in immunity (49).

The discovery of the role of Dscam in insect immunity may obscures the classical strict clarification between innate and adaptive immunity (13, 16, 40, 50). Accumulating evidence implicates Dscam might be involved in immunity against non-self-molecules in long-lived crustaceans, such as crab and shrimp. Dscam shows a typically rapid non-specific immune response to pathogen-associated molecular patterns such as LPS and beta-1,3-glucan (43, 44). However, in contrast to most innate immune factors, the induction of Dscam is not always an immediate response to stimulation by pathogens including viruses and bacteria (20, 27, 51). In this study, heat-inactivated bacteria were used for most of the experiment due to previously reported methods (16, 25, 32) and its low toxicity on primary cultured hemocyte, and we think Dscam will respond to live pathogens in the same manner since heat-killed bacteria still retain the key components and the bacterial clearance assay by using live bacteria confirm the function of Dscam on bacteria binding and phagocytosis promotion. Our study indicates that the role of Dscam in immunity is dictated not only by overall expression levels but also by the combination of Dscam isoforms. We found that some of the pathogen-induced Dscam isoforms that were highly induced after challenge with a particular pathogen showed significantly greater and more specific binding ability to that same pathogen, as well as efficient bacterial clearance. Although diversification is clearly critical for the generation of a sufficiently large pathogen receptor repertoire to allow discrimination among an equally large number of potential antigens, evidence that this level of specificity exists in pancrustacean immunity is limited (52). However, due to its extreme variability, the *Dscam* gene represents the only known system that could, at least theoretically, provide the required receptor diversity in pancrustaceans.

The topology of the eight Ig domains at the N-terminal of Dscam in *D. melanogaster* (21) has been elucidated by negative-staining electron microscopy. Averaged images of several isoforms obtained using this technique revealed multiple distinct configurations. By contrast, class averages of the N-terminal four Ig domains in the proteins revealed a horseshoe shape. Moreover, hemocytes of immunologically challenged *D. melanogaster* (13) and *Litopenaeus vannamei* (31) exhibited higher variability in the Ig2 and Ig3 domains of Dscam compared with those in the untreated controls, while few Ig7 variants were detected. For these reasons, we produced recombinant proteins covering only the Ig1–Ig4 region to study the immunological functions of *Es*Dscam. Interestingly, we found bacteria-specific binding activity is essential for Ig1–Ig4 domain-associated promotion of phagocytosis by soluble *Es*Dscam. Furthermore, although truncated recombinant *Es*Dscam proteins retained the ability to bind bacteria, the capacity to promote phagocytosis was abolished, possibly due to the absence of the region required for interaction with the phagocytic receptor on the hemocyte surface. Further investigations showed that RNAi-mediated knockdown of membrane-bound *Es*Dscam in hemocytes resulted in significant inhibition of soluble *Es*Dscam-mediated phagocytosis, which suggests a possible interaction between these two *Es*Dscam protein forms.

Importantly, parts of the Ig2 and Ig3 domains together form two surface epitopes (I and II) at either side of the conserved horseshoe structure, which are encoded partly by exon cluster 4 and partly by exon cluster 6 (21, 23). Epitope I is located on the composite surface that crucial for Dscam isoform interactions, while epitope II is located on the opposite face with the potential to interact with antigens. To study the possible interaction between membrane-bound and soluble *Es*Dscam, membrane-bound *Es*Dscam that share the same alternatively spliced exon as the soluble *Es*Dscam were knocked-down, and results revealed that the ability of membrane-bound *Es*Dscam to regulate the promoted phagocytosis by soluble *Es*Dscam was strictly alternatively spliced exon-specific. Furthermore, membrane-bound *Es*Dscam was found to be colocalized with soluble *Es*Dscam, and binding between Dscam isoforms was observed only in the same protein.

**Figure 9:**
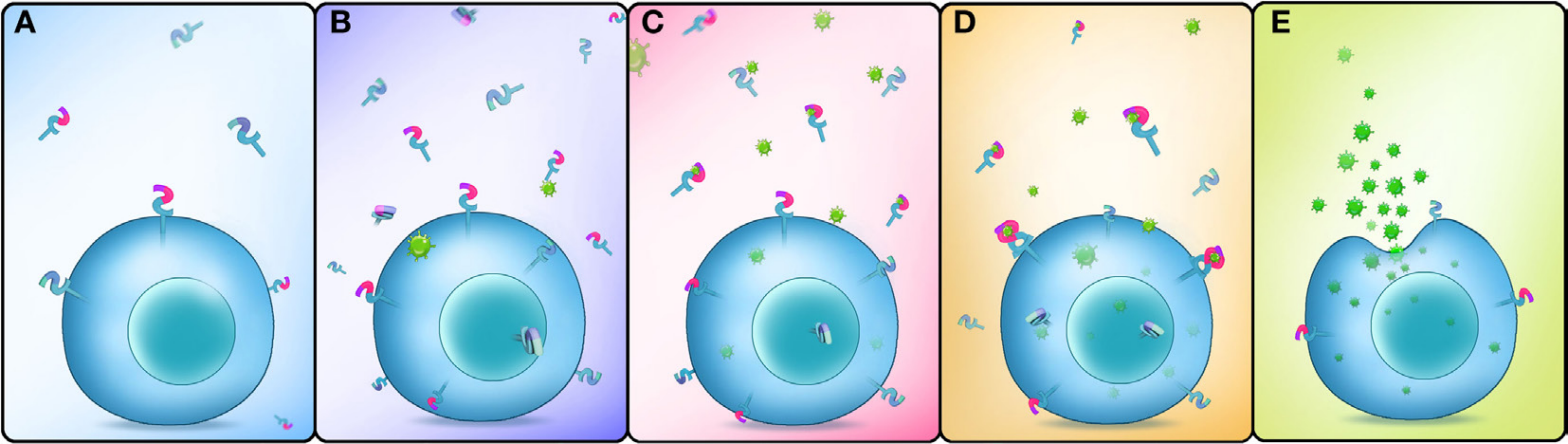
Schematic representation of Dscam-regulated pathogen-specific phagocytosis. **(A)** Lower concentrations of membrane-bound and soluble *Es*Dscam in normal hemocytes and cell-free hemolymph. **(B)** The expression of membrane-bound and soluble *Es*Dscam (including bacteria-specific induced and non-specific induced *Es*Dscam isoforms) is highly induced after bacterial infection. **(C)** Bacteria-specific induced soluble *Es*Dscam isoforms bind with high affinity to the original bacteria, while the other soluble isoforms show weak or no binding with the bacteria. **(D)** Bacteria-specific binding soluble *Es*Dscam isoforms interact with the membrane-bound *Es*Dscam containing the same extracellular region. **(E)** *Es*Dscam regulates pathogen-specific phagocytosis in crab hemocyte.

In conclusion, our study showed that *Es*Dscam is upregulated following bacterial challenge, and bacteria-specific *Es*Dscam isoforms are produced *via* alternative splicing. Subsequently, bacterial-induced specific soluble *Es*Dscam isoforms bind with the original bacteria and enhance phagocytosis by hemocytes *via* the membrane-bound *Es*Dscam that shares the same extracellular regions with the soluble *Es*Dscam that functions as the phagocytic receptor (Figure 9).

## Ethics Statement

This study was carried out in accordance with the recommendations of Ministry of Science and Technology of the People’s Republic of China on animal care guidelines. The protocol was approved by East China Normal University Animal Care and Use Committee (Protocol license number: AR2012/12017).

## Author Contributions

Conceived and designed the experiments: W-WL, QW, and X-JL. Performed the experiments: X-JL, LY, DL, and Y-TZ. Analyzed the data and wrote the paper: X-JL, QW, and W-WL.

## Conflict of Interest Statement

The authors declare that the research was conducted in the absence of any commercial or financial relationships that could be construed as a potential conflict of interest.

## Footnotes

^1^http://bioinf.cs.ucl.ac.uk/psipred/ (Accessed: April 16, 2015).

^2^http://weblogo.berkeley.edu/ (Accessed: May 12, 2015).

## Supplementary Material

Figure S1 The 3D structure of the *Es*Dscam protein (N-terminal Ig1–Ig8) and the location of epitopes I and II of the horseshoe configuration. **(A)** The 3D structure of the *Es*Dscam protein (N-terminal Ig1–Ig8). A 3D homology model of *Es*Dscam was generated using SWISS-MODEL (http://swissmodel.expasy.org/) (53) based on the crystal structure of Dscam from *Drosophila* (PDB ID: 3dmk.1.A) as a template (22). The 3D structure of *Es*Dscam was then visualized using the PyMOL molecular graphics system (54). The Ig domains are shown in different colors. **(B)** Structural comparison of modeled *Es*Dscam and *Drosophila melanogaster* Dscam. *Es*Dscam (red); *D. melanogaster* Dscam (green). **(C)** Location of epitopes I and II; epitope I of exon 4 (yellow); epitope II of exon 4 (red); epitope I of exon 6 (purple); epitope II of exon 6 (green). **(D)** Sequences of the 11 peptides used in the bacteria-binding assay. **(E)** The similarity and classification of exon cluster 4 and exon cluster 6. (*Left panel*) Exon cluster 4 was divided artificially into five groups, (*right panel*) exon cluster 6 was divided artificially into six groups.

Figure S2 Preparation of specific anti-*Es*Dscam antibody and loading control for various tissues. **(A)** Agarose gel electrophoresis (1% w/v) of the FNIII3–FNIII6 region of *Es*Dscam. Lane M1: 100–2,000 bp DNA maker. Lane 1: 1,455 bp cDNA amplification product. Lane 2: double digestion of recombinant plasmid pMD19-T–FNIII3–FNIII6 with *EcoR*I and *Xho*I restriction enzymes. Lane 3: double digestion of plasmid pET-28a with *EcoR*I and *Xho*I restriction enzymes. Lane 4: the PCR product of recombinant plasmid pET-28a–FNIII3–FNIII6. **(B)** Analysis of the recombinant *Es*DscamFNIII3–FNIII6 protein expression. Lane M2: 12–120 kDa protein markers. Lanes 1–4: recombinant protein expression under different induction conditions: 1 mM IPTG at 37°C; 1 mM IPTG at 30°C; 0.25 mM IPTG at 37°C, and 0.25 mM IPTG at 30°C, respectively. The arrow indicates the target protein. Lane 5: total proteins from uninduced cells. Lane 6: total proteins from induced cells harboring pET-28a (control). The molecular weight of induced protein is 62 kDa. **(C)** Purification of the recombinant *Es*DscamFNIII3–FNIII6 protein. Lane M3: 17–245 kDa protein markers. Lane 7: supernatant containing induced pET-28a–FNIII3–FNIII6. Lane 8: flow-through eluate of the supernatant. Lanes 9–17: protein purified from eluates containing different concentrations of imidazole. **(D)** *Es*Dscam antibody titers; preimmune serum was used as the control. **(E)** Western blot analysis of the specific binding of the purified recombinant protein with antiserum. Lanes 1–3: 10, 8, and 2 µg. **(F)** Protein loading control. (G)The specificity for detecting His antibodies used in immunofluorescence. Lane M: Protein marker. Lane 1: The total protein of hemocytes.

Figure S3 Exon 4 and exon 6 partitions of epitope I (magenta) and epitope II (cyan) in *Eriocheir sinensis*. In exon cluster 4, the 12 amino acids between the conserved 7Q and 18V were considered to belong to epitope I, and the 18 amino acids (approximately) after 43W were considered to belong to epitope II. In exon cluster 6, the eight amino acids after 15R were considered to belong to epitope I, and the eight amino acids before the conserved LLC motif were considered to belong to epitope II.

Figure S4 The expression and purification of r*Es*Dscam isoforms (Ig1–Ig4) and truncated r*Es*Dscam_4.24,6.19_ protein. **(A,B)** Recombinant expression plasmids: pET-28a-r*Es*Dscam**_4.24,6.19_**, pET-28a-r*Es*Dscam**_4.12,6.20_**, pET-28a-r*Es*Dscam**_4.6,6.9_**, pET-28a-rIg1-2, pET-28a-rIg2-3, and pET-28a-rIg3-4. The constructs were generated using the protocols described in Figure S2 in Supplementary Material. **(C,D)** Recombinant proteins expression and purification. **(C)** Lanes 1–4: recombinant protein expression under different induction conditions: 1 mM IPTG at 37°C; 1 mM IPTG at 30°C; 0.25 mM IPTG at 37°C, and 0.25 mM IPTG at 30°C, respectively. Lane 5: supernatant of induced pET-28a-rEsDscams. Lane 6: precipitation of induced pET-28a-rEsDscams. Lane 7: precipitation of induced pET-28a-rEsDscams. Lane 8: flow-through eluate of the precipitate. Lanes 9–18: protein purified from eluates containing different concentrations of imidazole. **(D)** Lane 1: total proteins from uninduced cells. Lanes 2–5: recombinant protein expression under different induction conditions: 1 mM IPTG at 37°C; 1 mM IPTG at 30°C; 0.25 mM IPTG at 37°C, and 0.25 mM IPTG at 30°C, respectively. Lane 6: precipitation of induced proteins. Lane 7: induced proteins in culture supernatants. Lane 8: induced proteins in culture supernatants. Lane 9: flow-through eluate of the supernatant. Lanes 10–19: protein purified from eluates containing different concentrations of imidazole.
