# Supplementary figures and images for: Pathogen-Specific Binding Soluble Down Syndrome Cell Adhesion Molecule (Dscam) Regulates Phagocytosis via Membrane-Bound Dscam in Crab
Source: Front Immunol. 2018 Apr 18;9:801. doi: 10.3389/fimmu.2018.00801 (PMC5915466; doi:10.3389/fimmu.2018.00801)

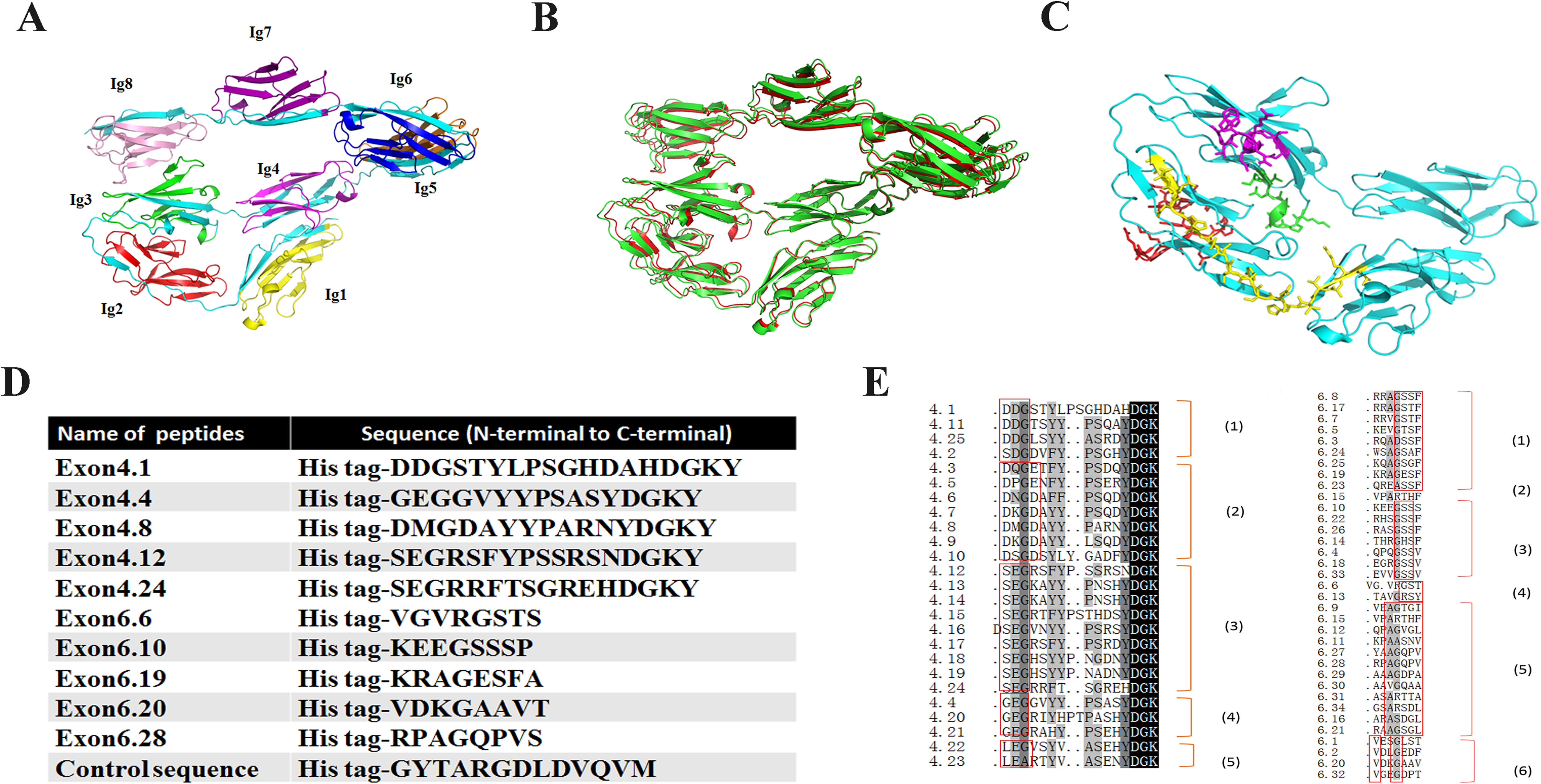

Supplement: Figure S1 — The 3D structure of the EsDscam protein (N-terminal Ig1–Ig8) and the location of epitopes I and II of the horseshoe configuration. (A) The 3D structure of the EsDscam protein (N-terminal Ig1–Ig8). A 3D homology model of EsDscam was generated using SWISS-MODEL (http://swissmodel.expasy.org/) (53) based on the crystal structure of Dscam from Drosophila (PDB ID: 3dmk.1.A) as a template (22). The 3D structure of EsDscam was then visualized using the PyMOL molecular graphics system (54). The Ig domains are shown in different colors. (B) Structural comparison of modeled EsDscam and Drosophila melanogaster Dscam. EsDscam (red); D. melanogaster Dscam (green). (C) Location of epitopes I and II; epitope I of exon 4 (yellow); epitope II of exon 4 (red); epitope I of exon 6 (purple); epitope II of exon 6 (green). (D) Sequences of the 11 peptides used in the bacteria-binding assay. (E) The similarity and classification of exon cluster 4 and exon cluster 6. (Left panel) Exon cluster 4 was divided artificially into five groups, (right panel) exon cluster 6 was divided artificially into six groups. [file Image_1.tif]

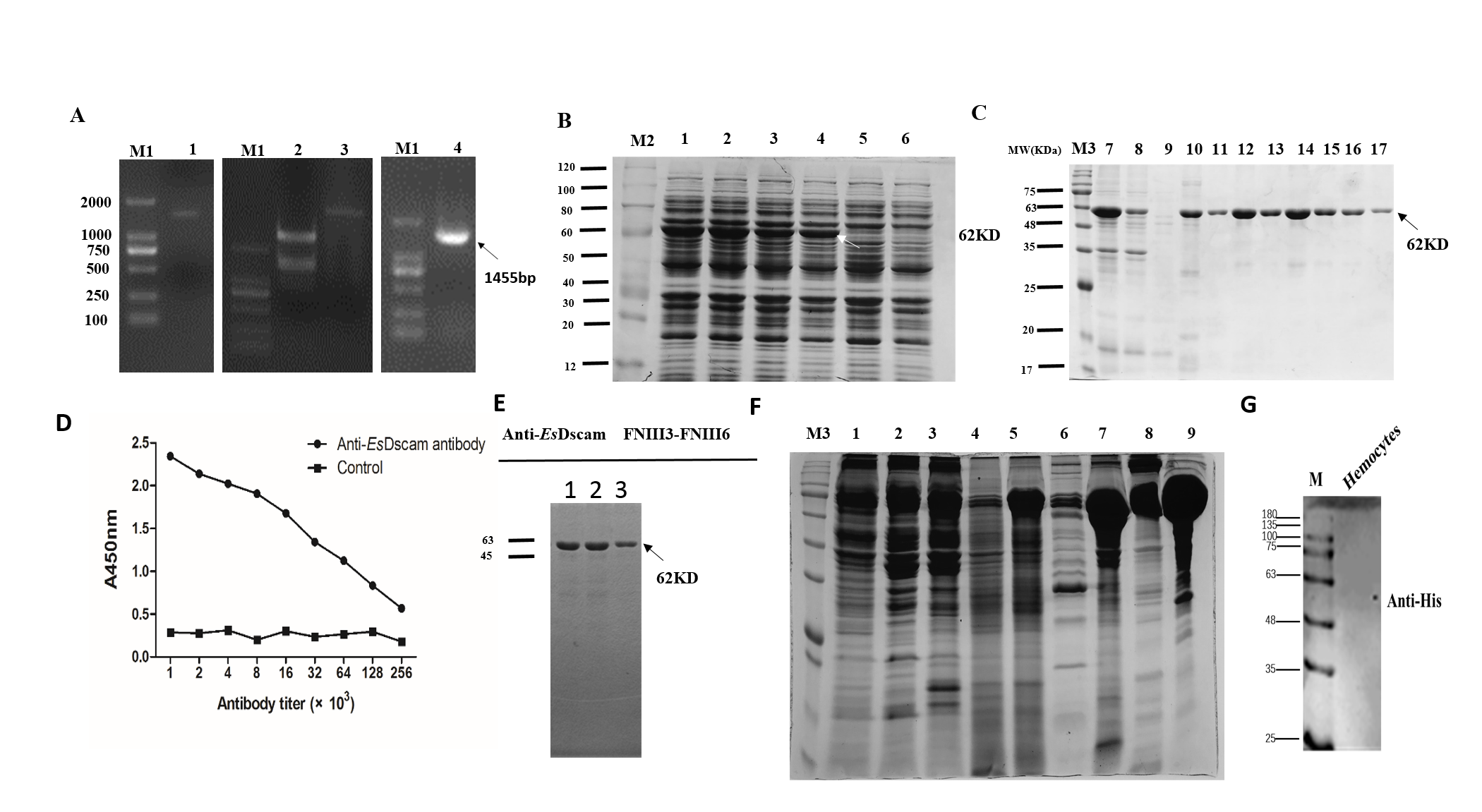

Supplement: Figure S2 — Preparation of specific anti-EsDscam antibody and loading control for various tissues. (A) Agarose gel electrophoresis (1% w/v) of the FNIII3–FNIII6 region of EsDscam. Lane M1: 100–2,000 bp DNA maker. Lane 1: 1,455 bp cDNA amplification product. Lane 2: double digestion of recombinant plasmid pMD19-T–FNIII3–FNIII6 with EcoRI and XhoI restriction enzymes. Lane 3: double digestion of plasmid pET-28a with EcoRI and XhoI restriction enzymes. Lane 4: the PCR product of recombinant plasmid pET-28a–FNIII3–FNIII6. (B) Analysis of the recombinant EsDscamFNIII3–FNIII6 protein expression. Lane M2: 12–120 kDa protein markers. Lanes 1–4: recombinant protein expression under different induction conditions: 1 mM IPTG at 37°C; 1 mM IPTG at 30°C; 0.25 mM IPTG at 37°C, and 0.25 mM IPTG at 30°C, respectively. The arrow indicates the target protein. Lane 5: total proteins from uninduced cells. Lane 6: total proteins from induced cells harboring pET-28a (control). The molecular weight of induced protein is 62 kDa. (C) Purification of the recombinant EsDscamFNIII3–FNIII6 protein. Lane M3: 17–245 kDa protein markers. Lane 7: supernatant containing induced pET-28a–FNIII3–FNIII6. Lane 8: flow-through eluate of the supernatant. Lanes 9–17: protein purified from eluates containing different concentrations of imidazole. (D) EsDscam antibody titers; preimmune serum was used as the control. (E) Western blot analysis of the specific binding of the purified recombinant protein with antiserum. Lanes 1–3: 10, 8, and 2 µg. (F) Protein loading control. (G)The specificity for detecting His antibodies used in immunofluorescence. Lane M: Protein marker. Lane 1: The total protein of hemocytes. [file Image_2.tif]

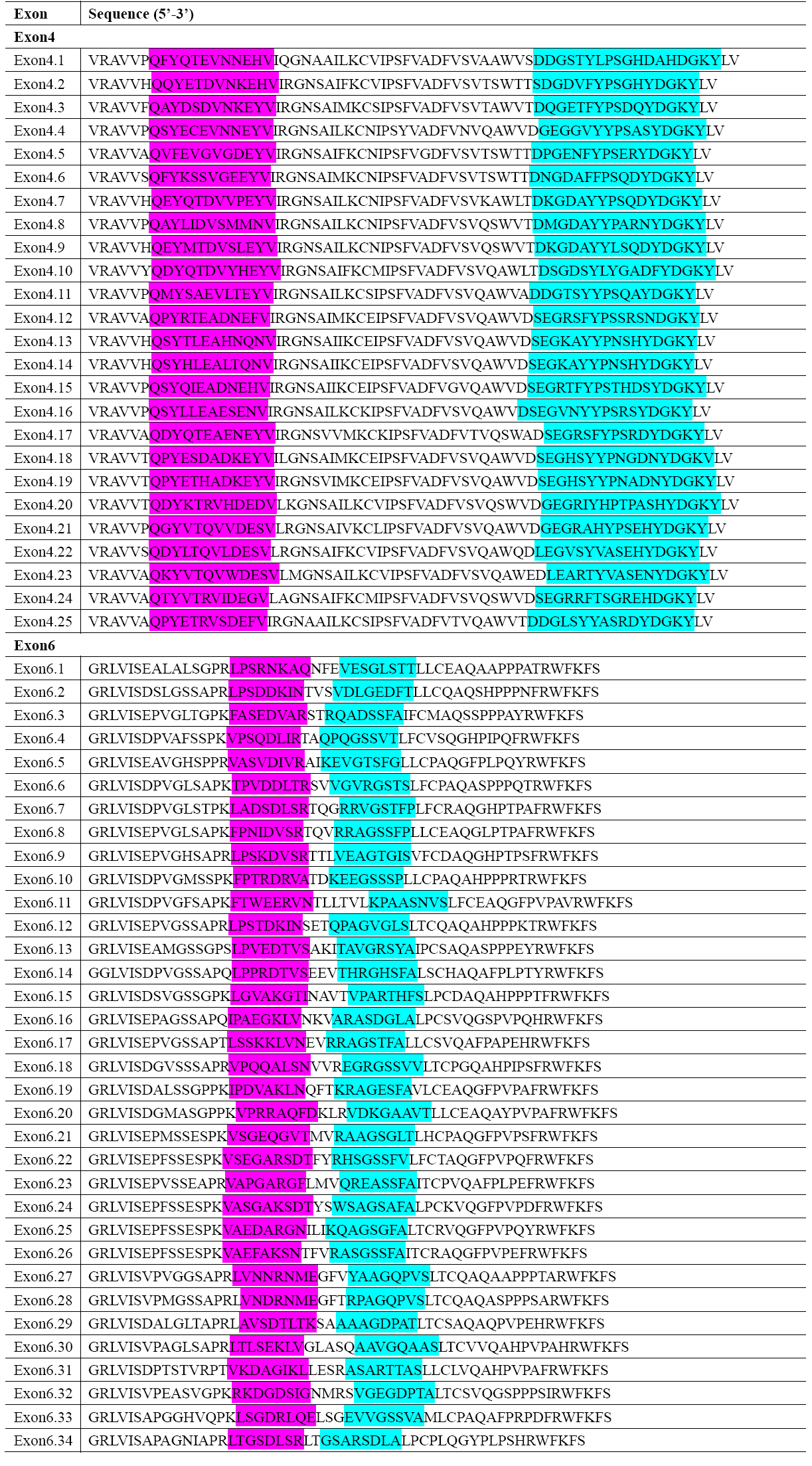

Supplement: Figure S3 — Exon 4 and exon 6 partitions of epitope I (magenta) and epitope II (cyan) in Eriocheir sinensis. In exon cluster 4, the 12 amino acids between the conserved 7Q and 18V were considered to belong to epitope I, and the 18 amino acids (approximately) after 43W were considered to belong to epitope II. In exon cluster 6, the eight amino acids after 15R were considered to belong to epitope I, and the eight amino acids before the conserved LLC motif were considered to belong to epitope II. [file Image_3.tif]

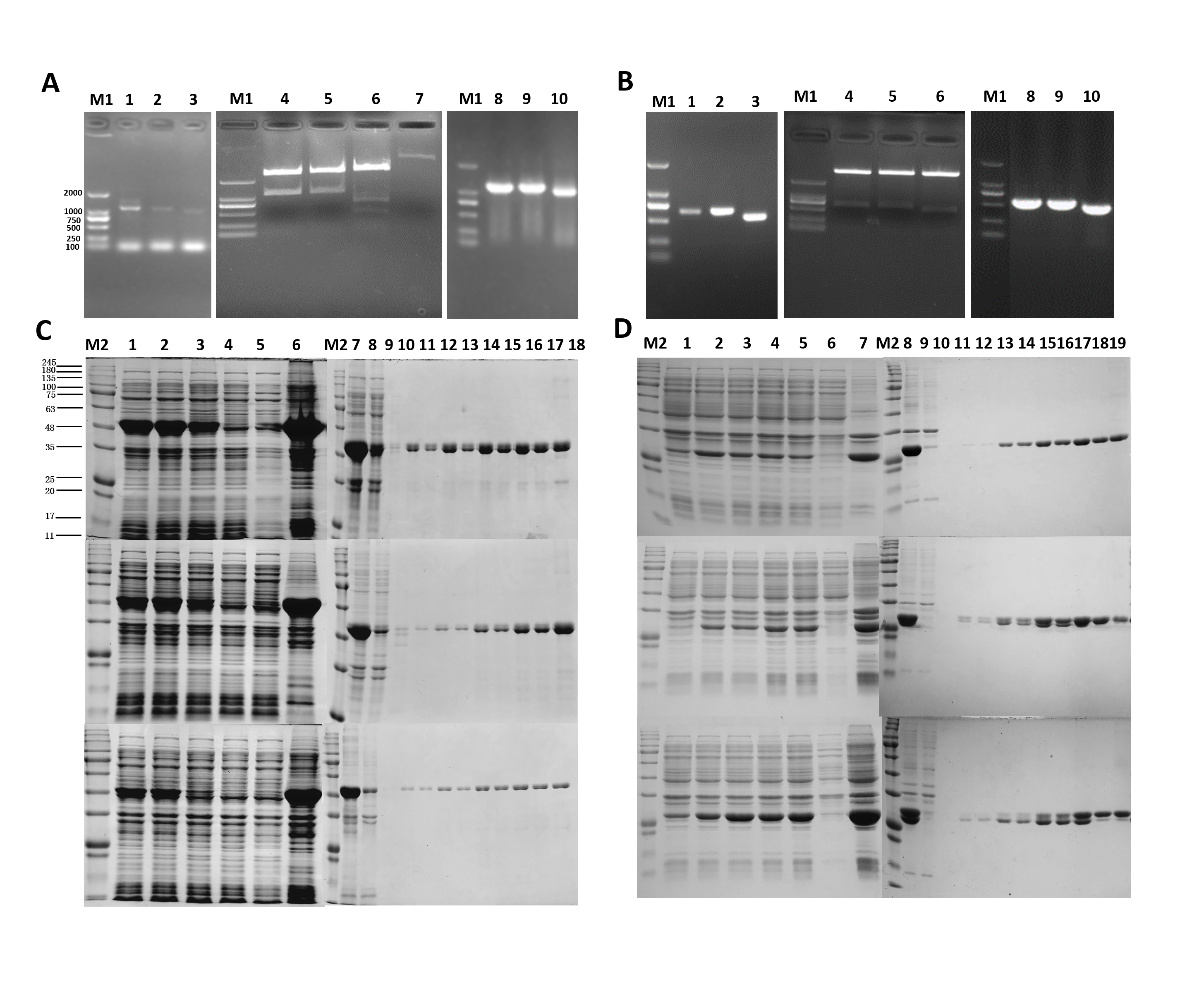

Supplement: Figure S4 — The expression and purification of rEsDscam isoforms (Ig1–Ig4) and truncated rEsDscam4.24,6.19 protein. (A,B) Recombinant expression plasmids: pET-28a-rEsDscam4.24,6.19, pET-28a-rEsDscam4.12,6.20, pET-28a-rEsDscam4.6,6.9, pET-28a-rIg1-2, pET-28a-rIg2-3, and pET-28a-rIg3-4. The constructs were generated using the protocols described in Figure S2 in Supplementary Material. (C,D) Recombinant proteins expression and purification. (C) Lanes 1–4: recombinant protein expression under different induction conditions: 1 mM IPTG at 37°C; 1 mM IPTG at 30°C; 0.25 mM IPTG at 37°C, and 0.25 mM IPTG at 30°C, respectively. Lane 5: supernatant of induced pET-28a-rEsDscams. Lane 6: precipitation of induced pET-28a-rEsDscams. Lane 7: precipitation of induced pET-28a-rEsDscams. Lane 8: flow-through eluate of the precipitate. Lanes 9–18: protein purified from eluates containing different concentrations of imidazole. (D) Lane 1: total proteins from uninduced cells. Lanes 2–5: recombinant protein expression under different induction conditions: 1 mM IPTG at 37°C; 1 mM IPTG at 30°C; 0.25 mM IPTG at 37°C, and 0.25 mM IPTG at 30°C, respectively. Lane 6: precipitation of induced proteins. Lane 7: induced proteins in culture supernatants. Lane 8: induced proteins in culture supernatants. Lane 9: flow-through eluate of the supernatant. Lanes 10–19: protein purified from eluates containing different concentrations of imidazole. [file Image_4.tif]
